# Protein diffusion in *Escherichia coli* cytoplasm scales with the mass of the complexes and is location dependent

**DOI:** 10.1126/sciadv.abo5387

**Published:** 2022-08-12

**Authors:** Wojciech M. Śmigiel, Luca Mantovanelli, Dmitrii S. Linnik, Michiel Punter, Jakob Silberberg, Limin Xiang, Ke Xu, Bert Poolman

**Affiliations:** ^1^ Department of Biochemistry, University of Groningen, Nijenborgh 4, 9747 AG Groningen, Netherlands.; ^2^ Department of Chemistry, UC Berkeley, Stanley Hall, Berkeley, CA 94720, USA.

## Abstract

We analyze the structure of the cytoplasm by performing single-molecule displacement mapping on a diverse set of native cytoplasmic proteins in exponentially growing *Escherichia coli*. We evaluate the method for application in small compartments and find that confining effects of the cell membrane affect the diffusion maps. Our analysis reveals that protein diffusion at the poles is consistently slower than in the center of the cell, i.e., to an extent greater than the confining effect of the cell membrane. We also show that the diffusion coefficient scales with the mass of the used probes, taking into account the oligomeric state of the proteins, while parameters such as native protein abundance or the number of protein-protein interactions do not correlate with the mobility of the proteins. We argue that our data paint the prokaryotic cytoplasm as a compartment with subdomains in which the diffusion of macromolecules changes with the perceived viscosity.

## Teaser

Native protein diffusion highlights heterogeneities in the bacterial cytoplasm.

## INTRODUCTION

The world of microbes holds many amazing examples of the complexity and completeness of the cell as a unit of life. The success of prokaryotes relies on a single, crowded cell to conduct the totality of its biochemical processes. Eukaryotic cells developed a plethora of membrane-bound compartments where certain metabolic reactions take place in separation from others. This compartmentalization allows for the coexistence of distinct physicochemical environments, which is beneficial or necessary for some biochemical reactions to occur. In archaeal and bacterial cells, such membrane-bound compartments are generally absent, with the exception of the periplasm in Gram-negative bacteria and, e.g., the anammoxosome in Planctomycetes (*1*). Thus, in most prokaryotes, the interior of the cell is one, uninterrupted solution—the cytoplasm. The replication and transcription of DNA, protein synthesis, and all the other cellular processes not taking place on lipid membranes or in the periplasm occur in this compartment.

**Fig. 1.**
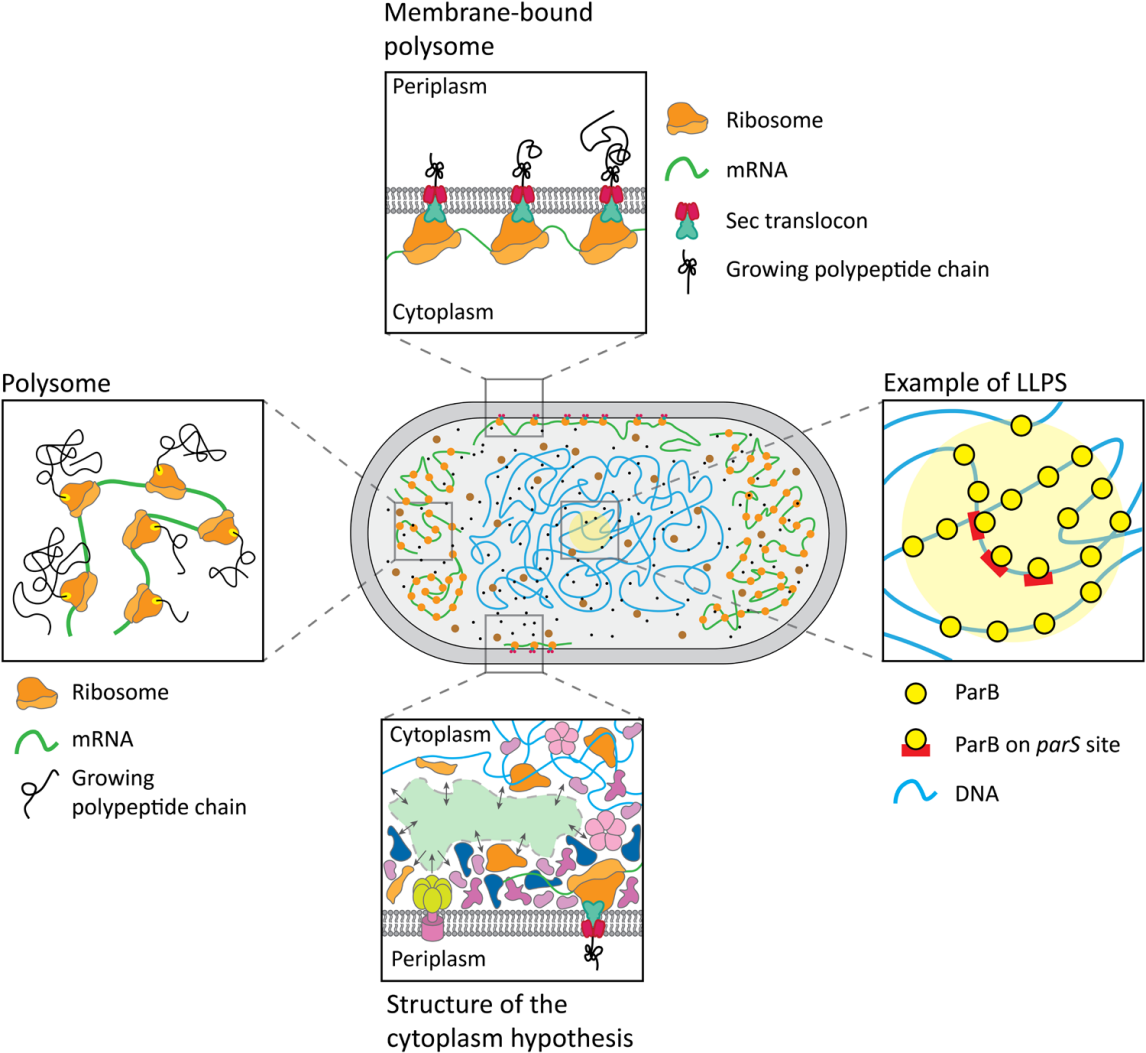
Schematic of the structure of the cytoplasm. The left, top, and right panels represent hyperstructures in the cytoplasm that could impair protein diffusion. The bottom panel shows the hypothetical undercrowded regions (green), where molecules can diffuse more rapidly, and overcrowded regions, where most of the macromolecules would be concentrated. The structure of the cytoplasm hypothesis is based on the notion that colloidal stability of the cytoplasm is brought about by hydrogen bonding with water molecules, excluded volume forces, and screened electrostatic interactions, which act over commensurate ranges of distances. The macromolecules would divide the interior of microspaces into dynamically crowded macromolecular regions and topologically complementary electrolyte pools.

The dimensions of prokaryotic cytoplasmic components range from the subnanometer scale for ions and metabolites to the micrometer scale for the chromosome, with the bulk of proteins and protein complexes in the range of a few to tens of nanometers (*2*, *3*). It has previously been shown that tested metabolites and native or heterologous proteins generally distribute uniformly in the cytoplasm of *Escherichia coli* (*4*, *5*). The cases of nonuniform distribution have been attributed to aggregation (*6*, *7*) or interactions of molecules with the large cellular components, which are the chromosome (*8*), the ribosomes (*9*), or the membrane (*10*) (Fig. 1). The chromosome and ribosomes can also be stably separated from each other, depending on whether mRNA is present to form polysomes, large structures of multiple ribosomes translating a single mRNA chain (*11*, *12*). Together with a recent study on the heterogeneous distribution of ribosomes (*13*), we estimate that the size cutoff for molecules that fit into the mesh of the nucleoid is as large as ribosomal subunits. Similarly, aggregated and/or misfolded proteins are squeezed to the cell poles (*14*). The situation is different in osmotically stressed cells: Hypertonic conditions can lower the size threshold for nucleoid occlusion to proteins as small as 27 kDa (*4*). Moreover, the bacterial cytoplasm has glass-like properties, which are most apparent under energy starvation (*15*, *16*). Metabolically active cells appear to have a more fluid cytoplasm (*15*). The reversible transition from a liquid-like state to a solid-like state has been proposed as a response mechanism to adverse conditions such as starvation (*17*), osmotic stress (*4*), and internal pH changes (*18*), both in bacteria and eukaryotes.

Another physical phenomenon that can take place in the crowded cytoplasm is the liquid-liquid phase separation (LLPS) (Fig. 1) (*19*, *20*). Pools of transiently interacting macromolecules can form membraneless compartments, such as droplets or less defined subdomains that are distinct from the surrounding lumen. By altering the local crowding or sequestering certain molecules, the phase-separated compartments can influence physiological processes, from enzymatic activity to the regulation of gene expression (*21*, *22*). The recent discovery of phase-separated compartments in bacteria points toward LLPS as yet another mechanism by which the prokaryotic cytoplasm could be compartmentalized. An excellent review of known and possible hyperstructures and their role in cell physiology is available (*23*, *24*), with many more on protein mobility under physiological and stress conditions (*25*–*28*).

The reports of heterogeneities in the distribution of molecules in cells fueled the hypothesis of the structure of the cytoplasm (*29*, *30*). Briefly, the attractive protein-protein interactions lead to the separation of the cytoplasm into denser, protein-rich subdomains and less crowded pools, where metabolites and proteins can diffuse faster (Fig. 1) (*31*). This view presents the cytoplasm as nonuniformly mixed, either for specific complexes (such as the polysomes) or generally for dynamic, protein-rich compartments intertwined with low-density domains. Here, we set out to challenge this and other hypotheses on the compartmentalization of the cytoplasm experimentally.

We probed the mobility of a diverse set of native *E. coli* proteins fused to the photoswitchable fluorescent protein mEos3.2 (*32*). The selected proteins vary in molecular weight, oligomeric state, abundance, and in the number of known interactions with other macromolecules. Moreover, the chosen targets have no reported interactions with the large cytoplasmic components such as DNA, RNA, ribosomes, or the membrane, and they are not known to be a part of LLPSs. To test the hypothesis that interactions required for the formation of the structure of the cytoplasm need time to evolve, we included two additional, non-native proteins that are homologs of *E. coli*’s TrxA present in the Gram-positive bacterium *Lactococcus lactis* and the archaeon *Haloferax volcanii*.

We chose the diffusion coefficient as a reporter of the physical state of the cytoplasm because of its dependence on the complex mass and sensitivity to the environment of the probe. Changes in crowding, molecular composition, or transition from the liquid to the glassy state of the cytoplasm should be reflected as a change in the lateral diffusion coefficient (*4*, *5*, *9*, *15*). The diffusion measurements allow us to test the hypothesis that proteins with a high number of interaction partners are more likely to participate in the structure of the cytoplasm than the ones with a low number of interaction partners. We have adjusted the recently developed single-molecule displacement mapping (SMdM) technique (*33*) to construct diffusivity maps of the *E. coli* cytoplasm at the scale of hundreds to tens of nanometers and a time resolution in the low millisecond range.

## RESULTS

### Target selection

To probe the structure of the cytoplasm, we selected a set of target proteins on the basis of the following criteria (fig. S1): The proteins are native to the organism and cytoplasmic, and they do not interact with the chromosome, mRNA, ribosomes, or the cell membrane. The molecular state, oligomeric weight, abundance, and potential interaction partners are known. We then selected a set of proteins varying in molecular weight, oligomeric state, and abundance. Last, we determined whether the proteins are suitable for C-terminal fluorescent protein tagging and overexpression, that is, targets that formed obvious aggregates at the cell poles were discarded from further experiments.

We chose *E. coli* BW25113, a widely studied derivative of the K-12 strain, as the host, and the genes were expressed from the arabinose promoter (*p_BAD_*). The subcellular localization and abundance of a substantial fraction of the *E. coli* proteins are known, and their interactions with other cellular components have also been documented (*34*). For most proteins, the oligomeric state is either confirmed experimentally or inferred from data on homologs. Moreover, quantitative, condition-dependent data on the proteome of *E. coli* BW25113 have been published (*2*, *3*).

We focus on investigating proteins with a relatively high copy number to prevent oversaturation of the native binding sites of the interacting partners. We reason that overexpressing proteins with a native copy number above 1000 per cell is more likely to produce representative data of the structure of the cytoplasm than overexpressing proteins that have a basal level of tens or hundreds of copies per cell. Relatively high expression is also necessary because SMdM requires a large number of foci to obtain diffusion maps of satisfactory resolution. The abundance data are from Schmidt *et al.* (*3*) for the growth conditions closest to our experimental setup (M9 media with glycerol as the carbon and energy source). Under these conditions, there are 573 proteins in *E. coli* with at least 1000 protein copies per cell, and these constitute, in total, 93.6% of the total protein content of the cell. We then cross-referenced the abundance data with binary interaction data from IntAct: 544 of the proteins had at least one known binary interaction with another *E. coli* protein, while 29 had none. Next, we excluded periplasmic, membrane, and ribosomal proteins and proteins with known or predicted interactions with the large cellular components (chromosome, mRNA, ribosomes, and the cell membrane) or involved in LLPS. We then manually selected 18 native *E. coli* proteins representing a wide range of molecular weights, oligomeric states, abundances, and loneliness values (table S1). Loneliness represents the ratio of abundance of the protein of interest to the sum of abundances of known interaction partners. The loneliness parameter does not represent the propensity of proteins to interact with their binding partners or the strength of the interaction; rather, it is an abstraction of the number of potential interactors per protein, and it is defined as

$$\text{Loneliness}=\frac{\text{Copy number of protein of interest per cell}}{\sum \text{Copy number of interactors per cell}}$$

For example, a protein of loneliness 10 has 10 copies per sum of all known interactors; a loneliness of 0.1 corresponds to one protein per 10 interaction partners.

### Single-molecule displacement mapping

**Fig. 2.**
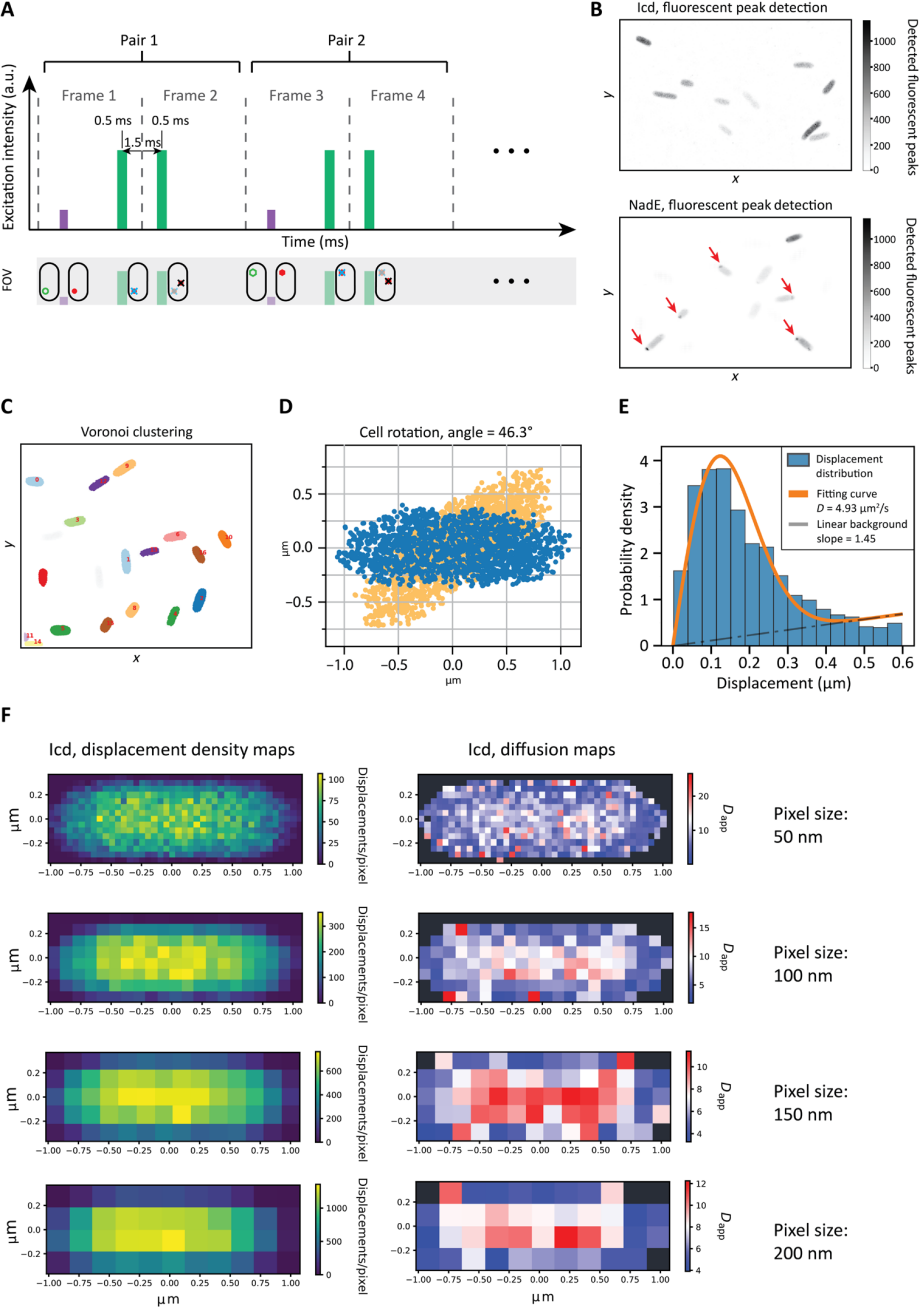
Overview of image acquisition and data processing. (**A**) Schematic of the SMdM method. The purple bar represents a short, low-intensity 405-nm laser impulse; the two green bars represent two consecutive short, high-intensity 561-nm laser pulses. The 405-nm pulse photoconverts mEos3.2 from green to red, which can then be excited by the 561-nm laser, and its emission is immediately detected. The second 561-nm pulse at the beginning of frame 2 excites again the same protein, allowing for a second observation of the same molecule, which has diffused to a new position over the time period of 1.5 ms. The laser intensity was chosen such that mEos3.2 typically bleaches after two 561-nm pulses, avoiding a misdetection in the following pair of frames. FOV, field of view; a.u., arbitrary units. (**B**) Fields of view depicted as a two-dimensional histogram where the intensity of each bin represents the number of fluorescent spots detected (see the “Single-molecule detection” section in Materials and Methods); examples of uniformly distributed (top) and aggregating (bottom) populations are shown. Zones of aggregation are visible as darker spots in the bottom panel. (**C**) An example of data clustering using Voronoi diagrams (*56*), which was applied on a field of view similar to that shown in (B). (**D**) Example of a cell before (orange) and after (blue) rotation. The point cloud describing the cell represents displacements, which are binned on the basis of their own starting position. (**E**) The diffusion coefficient is calculated for each pixel by fitting the data to a two-dimensional diffusion equation using maximum likelihood estimation, which results in a diffusion map. (**F**) Left: Displacement density maps at 50-, 100-, 150-, and 200-nm resolution. The color map represents the number of displacements per pixel. Right: Corresponding diffusion map obtained by fitting the data of each pixel with Eq. 1.

SMdM is an imaging technique that is based on the accumulation of a large number of displacements of particles at a fixed time step (Fig. 2A). Stroboscopic illumination of the sample with short, high-intensity laser pulses is timed so that the particles emit at the end of odd and the beginning of even frames. In this way, one loses the ability to track the particles over more than a single displacement, but the time step between the two recorded particle positions can be varied, which gives access to slow and fast diffusion regimes.

The top panel of Fig. 2B shows a typical field of view of cells uniformly expressing the gene encoding Icd fused to mEos3.2, which is representative for most of the tested constructs. For some proteins, we consistently observed aggregation of the proteins at the cell poles: In some cases, the aggregation occurred sporadically, whereas in others, dark foci were observed at the poles in most cells (Fig. 2B, bottom). We reason that the aggregation is the result of protein misfolding due to overexpression of the fusion construct and not a sign of native protein behavior. First, in most of the cells with signs of aggregation, we find foci of aggregates in one of the poles (Fig. 2B, bottom) instead of a symmetrical distribution of native large particles such as polysomes (*11*). Second, the foci were pushed to the cell poles and do not occupy the space between the replicated chromosomes in later stages of cell growth (*11*). Third, the aggregate structures were immobile over a period of 45 to 60 min. Hence, the cases shown in the bottom of Fig. 2B were excluded from the further analysis.

We analyzed each field of view as follows. The cells were automatically selected via Voronoi clustering and individually inspected and optimized for SMdM (Fig. 2C). First, each set of points representing a cell was rotated, so that the long axis of the cell was parallel to the *x* axis of the map (Fig. 2D). This was done to achieve better data density of the final diffusion map. The cells oriented in this way allow maximization of the number of fluorescent spots per pixel, as the longest flat feature of the cell can be aligned with the pixel grid. In this way, we obtain images with the displacement origin density for each cell (Fig. 2D). We then inspected each of the identified cells to see whether (i) the data are complete (the cell was not cut off by the edge of the field of view); (ii) the cell is not too close to other cells, to avoid clustering errors; (iii) there is no obvious cell division; and (iv) there is no visible protein aggregation. Cells that meet these criteria were further filtered on the basis of the total number of displacements (see the “Cell detection and rotation” section in Materials and Methods) and then mapped using a single-component, two-dimensional diffusion equation at the lowest feasible pixel size. By accumulating a large number of individual displacements, one can obtain enough data for fitting with an adjusted probability density function (PDF) equation (Eq. 1) and estimate the diffusion coefficient for a particular area (Fig. 2E). An explanation for the derivation of the equation is given in Materials and Methods

$$p(r,t)=\frac{1}{1-e^{-\frac{r_{\text{max}}^{2}}{4\mathrm{Dt}}}+\frac{k}{2}r_{\text{max}}^{2}}(\frac{2r}{4\mathrm{Dt}}e^{-\frac{r^{2}}{4\mathrm{Dt}}}+\mathrm{kr})\;0\leq r\leq r_{\text{max}}$$ (1)

The end result of SMdM is a diffusion map, the spatial resolution of which is determined by the number of accumulated displacements (Fig. 2F). To create diffusion maps, we varied the pixel size. Cells with low density of displacements require larger pixels and vice versa. Hence, we used pixel sizes of 50, 100, 150, or 200 nm, which yield maps of different spatial resolution (Fig. 2F, left). For the details of the experimental and analysis setup, we refer to Materials and Methods. Regardless of the cell size, displacement density, or diffusion coefficient (target protein), the center of the cell displays consistently a higher diffusion coefficient than the regions adjacent to the membrane or the poles (Fig. 2F, right); the pole regions are taken as ~20% of the cell length (see the “Cell area selection” section in Materials and Methods). In general, higher-resolution maps provide more spatial information but suffer from more variation in the calculated diffusion coefficients because of the lower number of displacements per pixel (*33*). The maps obtained by using a pixel size of 100 nm were then used to qualitatively inspect the diffusion of proteins. These maps are obtained by analyzing pixels that have at least 45 displacements, which ensures an SD of less than 15% (*33*).

### Quantitative implications of confinement

**Fig. 3.**
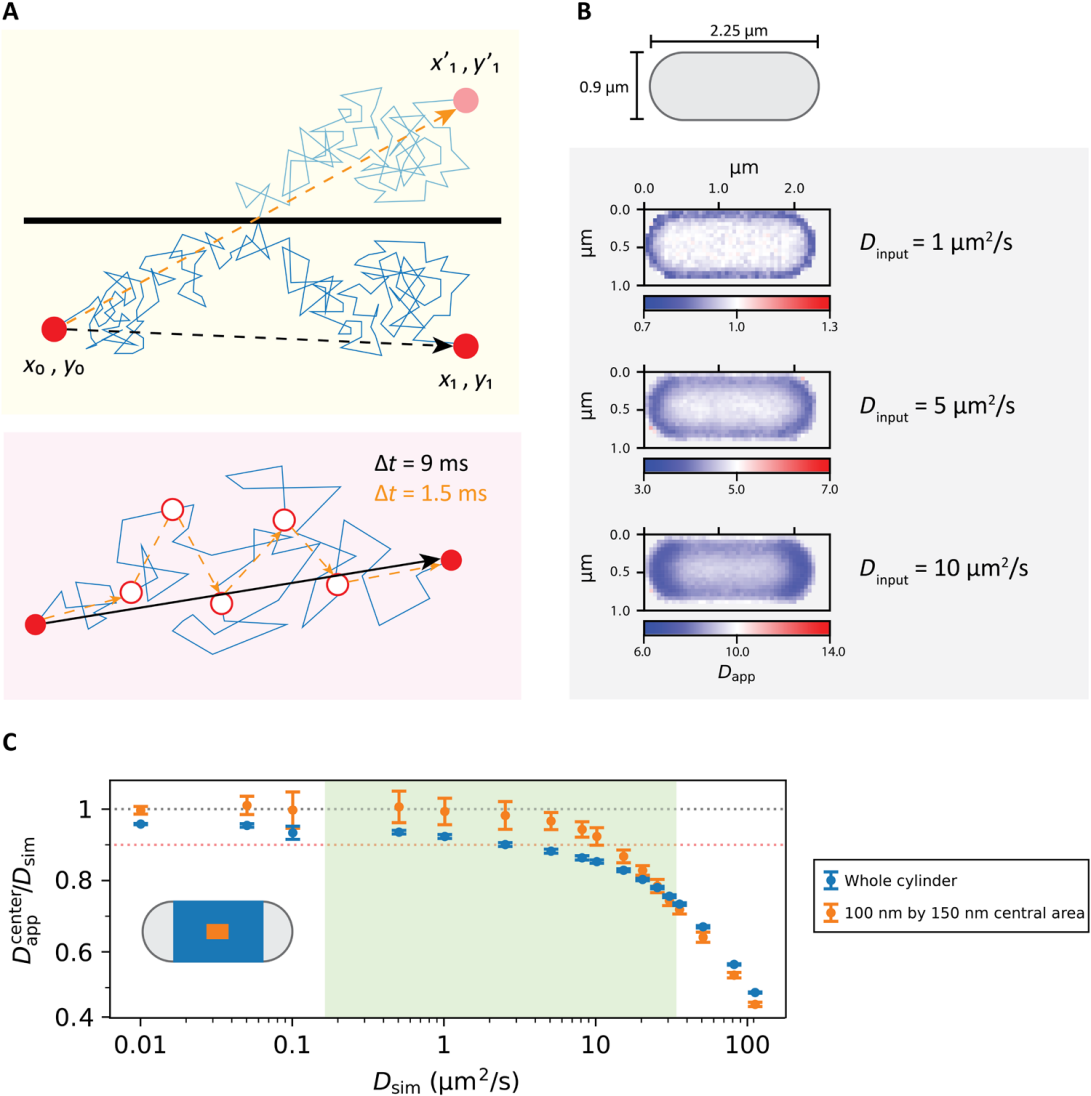
Implications of confinement for the analysis of diffusion data. (**A**) Top: Schematic of the impact of confinement on particle displacement at a fixed time resolution. The black arrow indicates the movement of the particle due to confinement. The orange arrow indicates the movement the particle would have made if it had not encountered a barrier. Bottom: Schematic of discretization of a particle movement trace from high to low time resolution. The blue line shows the actual trajectory of the particle. The black and orange arrows show the displacements that would be observed in a time frame of 9 ms and six consecutive time frames of 1.5 ms, respectively. (**B**) Top: Representation of an *E. coli* cell by a spherocylinder with dimensions used for Smoldyn simulations. Bottom: Diffusion maps of simulated spherocylinders with reflective surface containing particles diffusing at 10, 5, 1, 0.1, and 0.01 μm^2^/s. (**C**) Comparison of the dependence of the ratio of the apparent to input diffusion coefficient in a simulated spherocylinder when analyzing the centermost 100 nm by 150 nm area and the whole cylinder compartment. The orange dotted line represents a 10% decrease in the obtained diffusion coefficient compared to the input one, while the gray dotted line represents the ideal case in which the obtained diffusion coefficient is equal to the input one. The relevant range of diffusion coefficients for proteins is highlighted in green.

To understand the apparent diffusion slowdown close to the cell boundary (*35*), we determined the limitations of the diffusion mapping process. The basis for SMdM is the two-dimensional diffusion equation, which is continuous in both time and space. In our measurements, however, both time and space are discrete as we track the position of the molecules at a fixed time interval of 1.5 ms. Fitting the discrete displacements with the diffusion equation yields reliable data if the particles move randomly in an unobstructed space from the start to the end point of the displacement. This condition does not hold for particles near the cell membrane, and the observed displacement can be shorter than in the unobstructed area because of reflecting off of the boundary (Fig. 3A, top). Thus, it is possible that the apparent heterogeneities in the diffusion maps are caused by the confinement imposed by the cell membrane rather than an actual slowdown of the diffusing particle.

To investigate the basis for the apparent heterogeneities in the diffusion maps, we conducted in silico simulations of particles diffusing in spherocylinders with dimensions approximating those of an *E. coli* cell (Fig. 3B), using Smoldyn (*36*). The motion of particles with a predefined diffusion coefficient, time resolution, and compartment dimensions is simulated, and the model takes into account confining spaces with particles reflecting off of the defined boundaries. We then calculate the square root of the one-dimensional mean square displacement for the given time step. The software then picks a normally distributed random displacement for each axis, for each particle at each time step (*36*). In this way, we simulate a random walk, which is represented by the path obtained by the succession of random steps, which is a good approximation of the Brownian motion at short time steps (*37*). Given that our experimental time resolution was 1.5 ms, we choose to use 0.1 ms as the simulation time step. We ran our simulations for a total time of 2 s. Because random walk (and Brownian motion) is a Markov process, we could use the position of every particle at each time step as a starting position and the position of every particle 1.5 ms later as a final position (Fig. 3A, bottom). We then confined the simulated particles in spherocylinders with a length of 2.25 μm and a radius of 0.45 μm, which represented the median values of length and radius for all the analyzed cells (fig. S2). Precise length and width of all the analyzed cells can be found in table S2.

We simulated diffusion coefficients (*D*_sim_) ranging from 0.01 to 110 μm^2^/s. With the resulting particle positions at each time step, we constructed diffusion maps of the spherocylinders analogous to the SMdM measurements (Fig. 3B). We observe that the maps have the same characteristics of the cells analyzed by microscopy, with the cell center appearing to be a region of faster diffusion compared to areas near the compartment boundary, which is most notable in the region of the cell poles. This observation is an effect of the discrete time step used in mapping the diffusion coefficients, being too long to resolve the diffusive motion of this speed inside a compartment the size of an *E. coli* cell. In the 1.5-ms time span, particles close to the compartment boundary can travel distances sufficient to reach and reflect off of the boundary, which results in an underestimation of the diffusion coefficient. The apparent slowdown increases with the diffusion coefficient (Fig. 3B, bottom). Hence, reflections distort the results of SMdM, even when the underlying diffusion coefficient is uniform.

Instead of creating maps for all the simulated diffusions, we calculated the diffusion coefficients based on the displacements with starting points in the centermost 100 nm by 150 nm area of the cells. In this way, we only analyze displacements that are the least exposed to the confining effects of the compartment boundary. The analyzed areas of the simulated cells are able to reproduce the input diffusion coefficient *D*_sim_ up to a value of approximately 2.5 μm^2^/s (Fig. 3C). At values of *D*_sim_ higher than 10 μm^2^/s, the two-dimensional SMdM produces an apparent diffusion coefficient (*D*_app_) that underestimates the *D*_sim_ value by at least 10%. The underestimation of the *D*_sim_ value obtained from a larger area, like the whole cylinder part of the compartment (Fig. 3C), is even more pronounced than when the centermost 100 nm by 150 nm area is analyzed. Here, the *D*_app_ value does not reproduce the input diffusion coefficient *D*_sim_, underestimating it by at least 10% at a *D*_sim_ value of 2.5 μm^2^/s and by 15% at a *D*_sim_ value of 10 μm^2^/s (Fig. 3C).

The linear relation between the diffusion coefficient *D* and the lag time (or the time step), Δ*t*, allows us to imagine the hypothetical scenario where the acquisition time of SMdM is substantially faster than 1.5 ms. The obtained diffusion coefficient *D*_app_ in the areas near the cell boundaries is underestimated at input values of *D*_sim_ of 1 μm^2^/s or higher at Δ*t* value of 1.5 ms (see Fig. 3B, bottom); for accurate estimates of the mobility of a particle with a *D*_sim_ value of 10 μm^2^/s, a Δ*t* value of around 10 μs would be required, especially for the analysis of regions close to the cell boundaries. Reduction of Δ*t* value to the submillisecond time scale is not possible with conventional light microscopy cameras, given the brightness and photostability of photoactivatable fluorescent proteins, and the background fluorescence of the biological samples (*32*, *38*–*40*). However, given the linear dependence of *D* on Δ*t*, any reduction in acquisition time would improve the quality of the data in a predictable manner.

### Handling the limitations of SMdM in confined spaces

Despite the limitations of naive SMdM analysis of small cells, it is possible to draw conclusions from the data if the following are kept in mind. (i) The apparent diffusion coefficient will be lower than the actual diffusion coefficient (*D*_0_) of the tracked particles. The mismatch between *D*_app_ and *D*_0_ will depend on *D*_0_ and the location of the molecule in the cell. Hence, (ii) there will be patterns in the maps that will be the direct consequence of the confinement at the particular *D*_0_ and Δ*t* values. The patterns depend on the cell shape; therefore, (iii) we chose to compare diffusion coefficients obtained from cells of approximately the same dimensions (fig. S2). Last, (iv) the spatial resolution of the diffusion maps varies between the cells because of differences in data density. We therefore chose to analyze the acquired data for three arbitrary compartments— cell center and the two cell poles—which can be easily recognized even at low map resolutions. The two cell poles were automatically selected by taking the 20% of the total length of each cell and adding it to or subtracting it from its outermost left and right coordinates, respectively. In this way, we obtain a diffusion coefficient for a given protein that is least influenced by the confinement effects (cell center), and we are able to glimpse at the internal organization of a cell by comparing the diffusion coefficients of the cell center with those of the cell poles. Individual maps for every cell can be found in the Supplementary Materials. Confinement alone creates a difference between the apparent diffusion coefficient in the cell center and in the cell poles that will be dictated by the *D*_0_ of a given protein. While we are unable to estimate the *D*_0_ value accurately, we are able to compare the various proteins with their *D*_center_/*D*_pole_ ratios, exposing both trends and potential outliers. Therefore, we analyzed the cell center and cell poles separately (for details, see the “Cell area selection” section in Materials and Methods).

The *D*_app_^center^ values for the mean and SD reported in Table 1 are obtained from the analysis of the cell center; the actual numbers and the data for *D*_app_^poles^ are shown in table S3. Unless indicated otherwise, all *D*_app_ values are obtained by fitting the displacements with the adjusted PDF version of the two-dimensional diffusion equation (see the “Data fitting” section in Materials and Methods). The data are presented as the means of all selected cells for each construct, with errors representing the SDs. We did not find a fraction of slowly diffusing proteins (indicative of protein clustering or small aggregates), which would show up in SMdM as short displacements. We did not observe multiple diffusion coefficients within the three regions of the cells (cell center and cell poles); therefore, we analyzed our data using a single component fit.

**Table 1.** Lateral diffusion coefficients and data statistics of target proteins fused to mEos3.2. The given cell numbers represent single, nondividing cells without visible aggregation. The columns show the target protein, number of analyzed cells, abundance, loneliness, molecular weight, oligomeric state (1 - monomer, 2 - homodimer, 4 - homotetramer)’, and complex mass. The complex mass was calculated as the sum of the molecular weight of the monomeric protein plus mEos3.2 and multiplied by the oligomeric state number. The mean and SD of *D*_app_^center^ are shown in the last two columns. The UniProt ID is reported for all proteins, except for mEos3.2, for which the Fpbase ID is given. An extended dataset is given in table S1.

**Construct ID**	**UniProt ID**	**Protein name**	**Number of cells**	**Abundance (copies/cell)**	**Loneliness**	**MW (kDa)**	**Oligomeric state**	**Complex mass (kDa)**	***D*_app_^center^**	
									**Mean**	**SD**
1	VUXFR*	mEos3.2	30	–	–	25.7	1	25.7	11.4	1.6
3	P00934	ThrC	31	11,109	0.350	47.1	1	72.8	7.8	1.1
8	90AC62	GrxC	26	6,170	89.400	9.1	1	34.8	10.3	1.3
9	P05793	IlvC	22	29,065	36.200	54.0	4	318.9	2.9	0.5
11	P08997	AceB	28	8,308	10.400	60.2	1	85.9	6.9	1.1
12	P0A6A8	AcpP	38	28,863	0.120	8.6	1	34.3	9.6	1.6
13	P0ACC3	ErpA	22	3,460	0.100	12.1	2	75.5	7.8	1.1
15	P0AA25	TrxA	33	18,242	0.025	11.8	1	37.5	8.7	1.8
16	P07813	LeuS	20	1,505	0.005	97.2	1	122.9	4.1	0.8
19	P08200	Icd	23	24,591	1.020	45.7	2	142.8	5.0	0.9
15_hvo	A0A558GCJ2	TrxA2_hvo	23	–	–	12.1	1	37.8	8.1	1.2
15_lla	A0A089XQE8	TrxA_lla	24	–	–	11.7	1	37.4	6.6	1.3

### Correlation between diffusion coefficient and target parameters

**Fig. 4.**
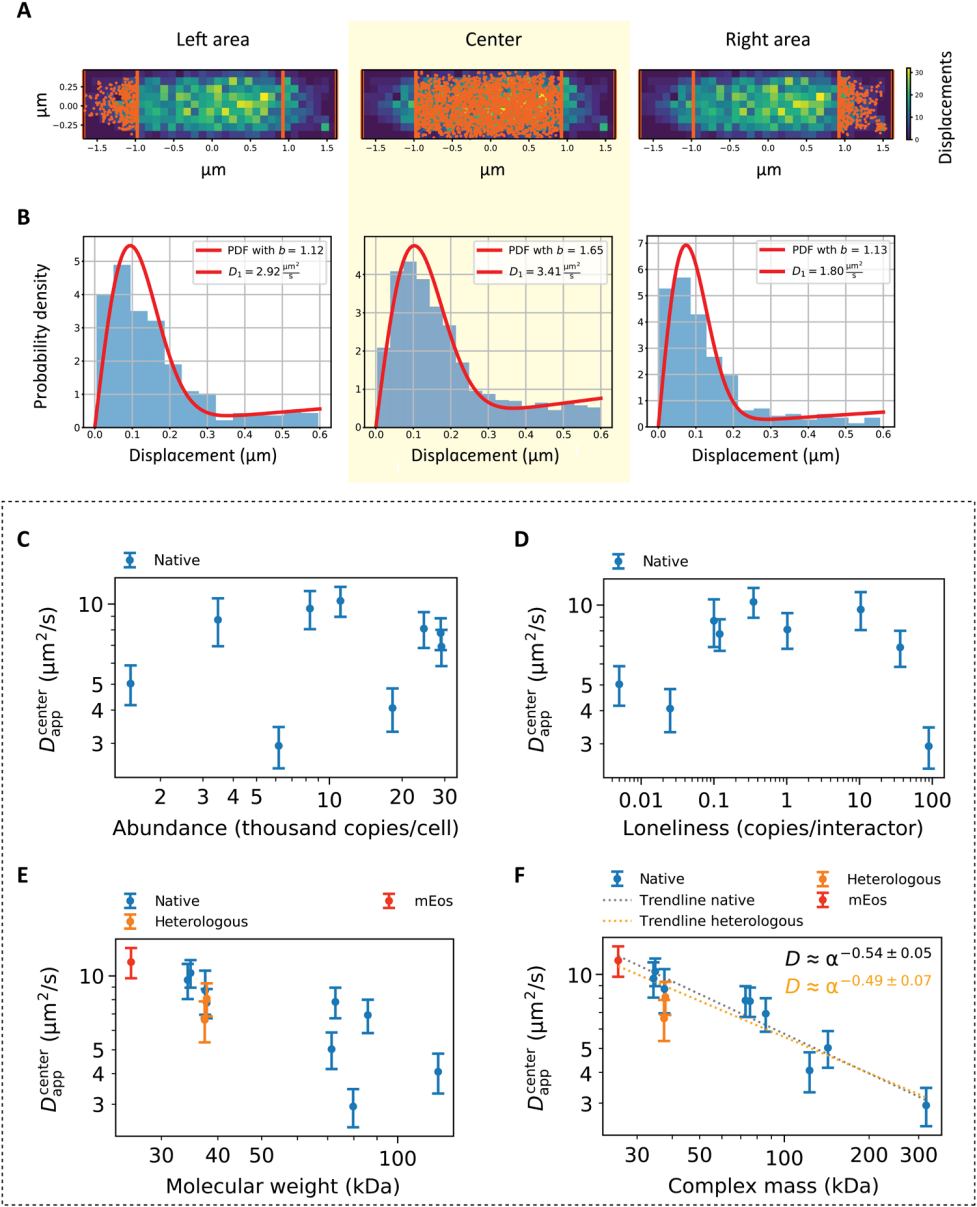
Protein diffusion in the cell center. (**A**) Cells were divided in three main regions: the cell center and the cell poles. The displacements belonging to each region were then analyzed separately. (**B**) Fit of the displacements represented in (A). The fits of the displacements belonging to the cell center (highlighted in yellow) were used for the analyses represented in (C) to (F). (**C** to **F**) Dependence of the *D*_app_^center^ on (C) the native protein abundance, (D) loneliness, (E) molecular mass of the monomeric unit (that is, the sum of the monomeric protein target’s molecular weight and that of mEos3.2), and (F) complex molecular mass, which takes into account the oligomeric state. Native proteins are indicated in blue, TrxA homologs are indicated in orange, and mEos3.2 is indicated in red. The gray trendline in (F) is obtained by calculating the dependence of the diffusion coefficient on the complex mass without considering the heterologous proteins, while the orange trendline is obtained when the heterologous proteins are included. Abundances of native protein expression were used for (C) and (D); hence, the heterologous proteins are missing from the graphs.

We analyzed the diffusivity of the native proteins in the cell center region (Fig. 4, A and B) as a function of the selection parameters in cells with a consistent shape (fig. S2). Previously reported values for free diffusion of wild-type green fluorescent protein (GFP) in exponentially growing *E. coli* (*9*) are consistent with the values observed in this study for freely diffusing mEos3.2, which shares molecular weight and physical chemical properties with GFP (*32*).

We find no apparent dependence of the diffusion coefficient on protein abundance (Fig. 4C) or loneliness (Fig. 4D). The dependence of the diffusion coefficient on the molecular weight (Fig. 4E) of the diffusing particle is far less scattered when the oligomeric state is taken into account (Fig. 4F). Note that in our calculations, we assume that all subunits are tagged with mEos3.2. Our conclusions are supported by the calculation of Spearman’s rank correlation coefficient (*r*; see the “Statistical analyses” section in Materials and Methods), which indicates good correlation between datasets if the result is higher than 0.8 for positive correlation or lower than −0.8 for negative correlation. Analyzing the dependence of the diffusion coefficient on protein abundance results in *r* = −0.07; a value of *r* = −0.05 is observed for loneliness and *r =* −0.8 with *P* < 0.01 for molecular weight. Most significant is the dependence of diffusion coefficient on complex mass, which was calculated as the sum of the molecular weight of the monomeric protein plus the fluorescence reporter and multiplied by the oligomeric state number. The dependence on complex mass has *r* = −0.88, indicating a very strong correlation with *P* << 0.01. We note that the heterologous TrxA proteins are slightly offset compared to the trendline (Fig. 4D), but we find similar *r* and *P* values when these non-native proteins are excluded. We fitted the dependence of the diffusion coefficient on complex mass with a power law relationship *D* = α*M*_complex_^β^, where *M*_complex_ is the complex mass and α and β are fitting parameters (Fig. 4F), and analyzed the residuals (fig. S3). We observe no correlation of the residuals with the complex mass as parameter. We find that the diffusion coefficient of the native *E. coli* proteins scales proportionally to the complex molecular mass according to a power law: *D* ≈ α*M*_complex_^−0.54 ± 0.05^. We also performed multiparametric Spearman’s rank correlation coefficient analysis between all the considered variables (fig. S4), and we do not observe correlation between abundance, loneliness, and molecular weight, indicating that the outcome of the correlation between each of these variables and the diffusion coefficient is not influenced by any of the other variables.

### Contribution of surface charge to protein mobility

**Fig. 5.**
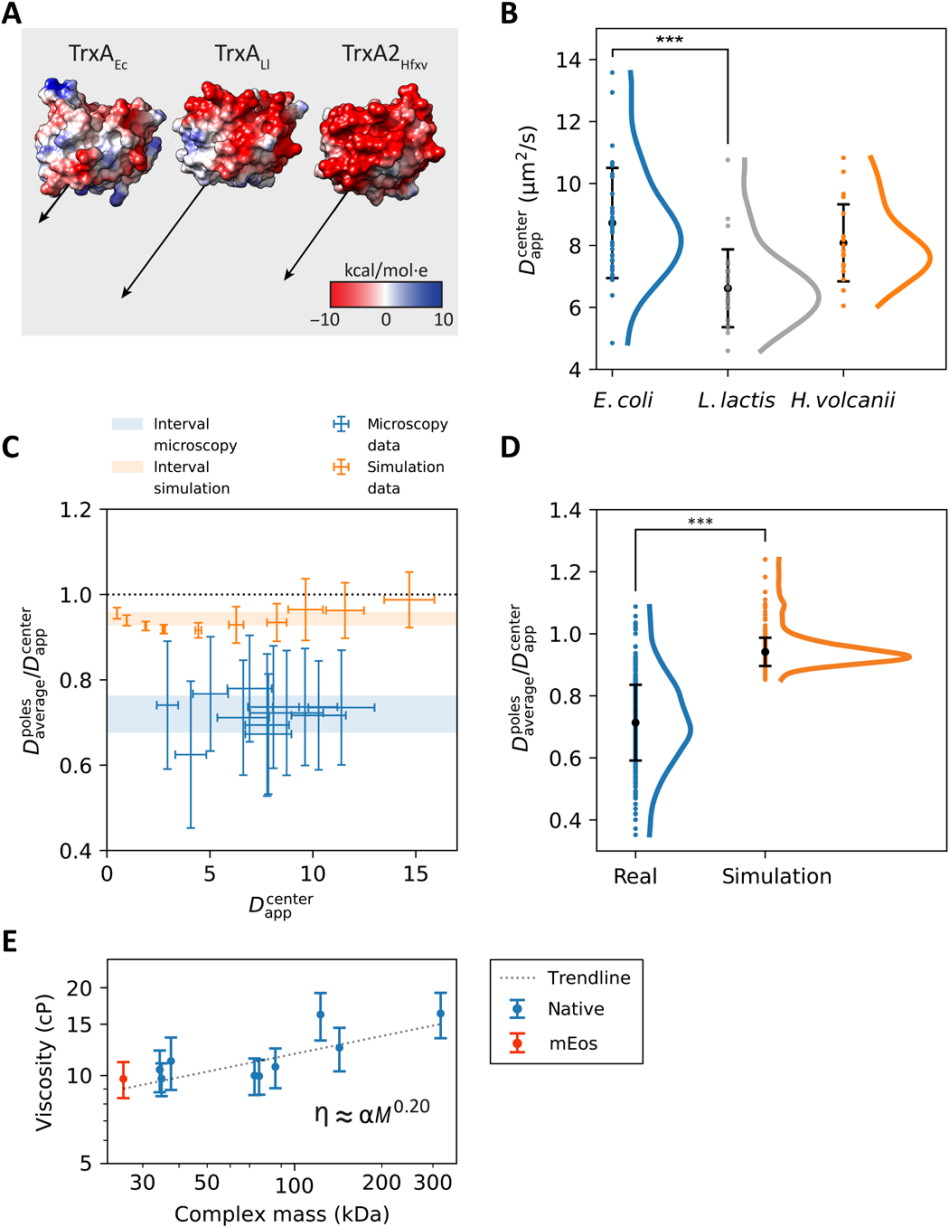
Physical chemical effects on protein diffusion. (**A**) Molecular models of the three homologous thioredoxin proteins from *E. coli*, *L. lactis*, and *H. volcanii*, based on the Protein Data Bank structures 3DXB, 2O87, and 6KIL for *E. coli*, *L. lactis*, and *H. volcanii* and named TrxA_Ec_, TrxA_Ll_, and TrxA_Hfxv_, respectively. The Coulombic surface charge is depicted using red and blue coloring as the negative and positive charge, respectively. Dipole moments are depicted as black arrows. (**B**) Scatterplots of the *D*_center_ value of the three TrxA proteins. The means are shown as black dots; the error bars represent the SDs. The curves next to the scatterplots are obtained via kernel density estimation. Statistical significance indicated with asterisks. (**C**) Relative slowdown of diffusion at the cell poles for both experimental SMdM and simulated data. The average of the two *D*_app_^poles^ for each cell is divided by the *D*_app_^center^ and plotted against the *D*_app_^center^. The blue and orange areas represent the means ± SD of all the ratios for the microscopy and the simulated data, respectively. The black dotted line represents the case when there would be no slowdown at the cell poles. (**D**) Scatterplots of the data presented in (C). The means are indicated by black dots, with black bars representing the SDs. The curves next to the scatterplots are obtained via kernel density estimation. Statistical significance indicated by asterisks. (**E**) Intracellular perceived viscosity as a function of the molecular weight of protein complexes. The trendline is obtained by fitting the formula η = α*M*^0.15^.

To analyze the possible effects of protein-protein interaction on the diffusion of closely related proteins with nearly identical mass, we analyzed three homologous thioredoxins, which are the Trx proteins from *E. coli*, *L. lactis*, and *H. volcanii*. We reasoned that the native *E. coli* thioredoxin might have a lower diffusion coefficient due to a larger number of interaction partners than the heterologous proteins. We tested the distributions of the diffusion coefficients of the three proteins for normality using a Shapiro-Wilk test and observe that the three datasets did not appear to be normally distributed, with *P* < 0.05. We find that their mean diffusion coefficients differ from one another, with the most significant difference between the native thioredoxin and the homolog from *L. lactis*, for which the Mann-Whitney *U* rank test (see the “Statistical analyses” section in Materials and Methods) results in *P* << 0.01 (Fig. 5B). We do not confirm our hypothesis that the native protein may have a lower mobility due to a larger number of potential unique protein-protein interactions, which are 87 for TrxA_Ec_ with a relatively low loneliness of 0.025 (or about 40 interaction partners per TrxA_Ec_ molecule). The three proteins have a very similar molecular mass, and the apparent slowdown may come from nonspecific interactions with the native cytoplasmic components. Inspecting the surface charge distribution of TrxA_Ec_, TrxA_Ll_, and TrxA_Hfxv_ shows that the native protein has the negative and positive charges interspersed relatively uniformly throughout the protein surface (Fig. 5A and fig. S5). The *L. lactis* and *H. volcanii* thioredoxins show far more anionic surfaces, which are also reflected in a much higher dipole moment, which are 236, 477, and 425 Debye for TrxA_Ec_, TrxA_Ll_, and TrxA_Hfxv_, respectively (Fig. 5A). TrxA_Ll_ has the lowest diffusion coefficient (Fig. 5B) and, owing to a highly anionic surface opposite a neutral-positive patch, the largest dipole moment (Fig. 5A). We do not find a significant correlation between the diffusion coefficient and the dipole moment, and we conclude that the differences in surface polarization of the proteins may not solely explain the variation in diffusion coefficients (*9*).

### Analysis of protein diffusion at the cell poles

We then compared the diffusion at the poles relative to the diffusion at the cell center, which is possible because the cell shape is similar for all the investigated proteins (fig. S2). Hence, the characteristics of macromolecular confinement are considered the same for each protein. We calculated the ratio between the diffusion coefficient measured at the poles and at the center of the cell for each analyzed protein and for each simulated diffusion coefficient, and we observe that, in both cases, those ratios were localized around constant values: 0.71 for microscopy data and 0.94 for simulated data (Fig. 5C). Therefore, the diffusion of each protein is slower at the poles than in the center of the cell. Next, we analyzed the data collectively and compared the ratios between the diffusion coefficient measured at the poles and center for all cells expressing the protein constructs and for all the simulated diffusions (Fig. 5D).

We see that the diffusion coefficients from the pole regions are offset by a similar fraction for all the proteins, which is much more prominent for the experimental SMdM than for the simulated data (Fig. 5D). Simulated data were obtained from the movement of particles at different diffusion coefficients, ranging from 0.5 to 20 μm^2^/s, in spherocylinders of various dimensions with diameters ranging from 0.41 to 2.34 μm and a length from 1.1 to 3.64 μm, which represent the minima and the maxima for cell width and cell length, respectively, in the experimental dataset. In this way, we obtain some degree of heterogeneity in the simulated population. We tested both distributions for normality using a Shapiro-Wilk test. The microscopy data are normally distributed with *P* > 0.05, while the simulated data appear to be non-normally distributed with *P* << 0.05. When analyzing the simulated data with kernel density estimation, we observe an extended tail at the higher *D*_app_^poles^/*D*_app_^center^ ratios, with values above 1 (Fig. 5D), which reflects the higher diffusion coefficients (Fig. 5C). We reason that these high ratios are caused by simulating fast diffusion in the shorter cells: A particle originating from one pole and diffusing with a high diffusion coefficient would end up further away from its origin than a particle originating from the cell center and bouncing against one of the cell poles. In this scenario, the ratio between diffusion measured at the poles and diffusion at the cell center is characterized by values higher than 1. The SMdM microscopy data show a bigger spread compared to the simulation data, and the *D*_app_^poles^/*D*_app_^center^ ratios are significantly lower for the experimental than simulated data; the Mann-Whitney *U* rank test (see the “Statistical analyses” section in Materials and Methods) shows *P* << 0.01. Thus, the lateral diffusion of proteins is substantially slower at the poles compared to the cell center.

## DISCUSSION

We performed single-molecule diffusion measurements using the recently developed SMdM technique (*33*). SMdM has a number of advantages over conventional single-particle tracking and ensemble diffusion measurements such as FRAP (fluorescence recovery after photobleaching). The short illumination pulses allow for precise determination of the positions of fast-moving small proteins and large, relatively immobile particles at the same time, which enables investigating proteins potentially involved in formation of dynamic complexes. SMdM has a spatial resolution high enough to confidently section the small prokaryotic cells into zones of interest, allowing to obtain information on diffusion in prokaryotes at a level of detail never achieved before.

### Confinement influences the SMdM readout

Application of the SMdM to *E. coli* cells reveals the capability of the method to resolve the substructure of the cytoplasm despite the challenge of having a small confined compartment. We show by simulations and experimental analyses that the relatively long lag time of the experimentally measured displacements, the high diffusion coefficients of the cytoplasmic components, and the small size of the cells result in an underestimation of the apparent diffusion coefficients. The magnitude of this underestimation depends on the actual diffusion coefficient of the moving particle and is dependent on the compartment shape, with the apparent slowdown increasing near the cell boundary. However, it is possible to obtain important and interpretable data on the physical state of the cytoplasm. We obtain diffusion maps with a resolution of 50 to 200 nm, depending on the data density and the length of the experiment. While the *D*_app_ values are skewed by the macromolecular confinement, all the measurements have in common the similar cell geometry, allowing the data to be compared between the different cells, constructs, and areas within the cells.

### Protein diffusion scales with the mass of protein complexes

From the analyses performed on selected target proteins, we find that the diffusion coefficients scale with the complex molecular mass, that is, the mass of the tagged polypeptide chain multiplied by the oligomeric state, and not with abundance or loneliness. In addition, for three homologous proteins with different surface charge distribution and dipole moments, we observe significant differences in the apparent diffusion coefficient between the *E. coli* and *L. lactis* TrxA. The TrxA_Ll_ and TrxA_Hfxv_ proteins have similar highly anionic surfaces, yet they differ in cytoplasmic mobility. We conclude that nonspecific electrostatic interactions with other cell components alone cannot explain the variation in diffusion of TrxA proteins, unlike what has been found for cationic fluorescent proteins (*9*).

In exponentially growing *E. coli* cells, at the spatial resolution of around 50 to 200 nm and time resolution of 1.5 ms, we do not observe dynamic subdomains in the central region of the cytoplasm, but we do find a slowdown of protein diffusion at the cell poles. The aggregation of SlyD, LeuB, OsmC, Ndk, NadE, MetK, and AceE is most likely an artifact of the protein overexpression, rather than a physiological substructure of the cytoplasm, because the structures are immobile and vary from cell to cell, unlike the megadalton polysomes (*11*). Last, SMdM of proteins in small compartments such as prokaryotic cells requires accumulation of data over time (up to an hour, with an average acquisition time of ~30 min); hence, dynamic structures that form and disassemble on a very short time scale will not be detected. We also note that the cytoplasm reorganizes into different physical states under adenosine 5′-triphosphate (ATP) depletion or specific stress conditions as seen in prokaryotes and eukaryotes (*4*, *15*, *17*, *18*), conditions that have not been probed in this study. In this context, it is worth noting that ATP at millimolar concentration has the ability to prevent the formation of and dissolve previously formed protein aggregates (*41*).

### The cell cytoplasm behaves as a dilatant fluid

The observed dependence of the diffusion coefficient on the complex molecular mass has a power law with *D* ≈ α*M*_complex_^−0.54^, where α is a scaling factor and *M*_complex_ is the mass of the native protein complex. This dependence is in line with previous observations (*42*, *43*), but the slope deviates from the value predicted by the Einstein-Stokes equation

$$D=\frac{k_{B}T}{6\pi \eta r}$$ (2)

where *k*_B_ is the Boltzmann constant, *T* is the absolute temperature, η is the viscosity of the solvent, and *r* is the radius of the diffusing particle. According to the equation, the relationship between diffusion coefficient and complex molecular mass is *D* = α*M*_complex_^−0.33^ (see the “On cytoplasmic viscosity” section in Supplementary Text), assuming that the proteins are globular and not interacting with other particles in the solution. We argue that the discrepancy between the observed and the theoretical value cannot be attributed solely to shape differences between the target proteins. In that case, we would not have found the relationship with the complex molecular mass. A similar argument could be made for surface charge. If deviations from the Einstein-Stokes equation were due to differences in protein charge, then we would have lost the observed relationship. Most likely, the stronger-than-predicted dependence on molecular mass reflects the high macromolecular crowding of the cytoplasm and the collisions with other macromolecules (here, we introduce the term “macromolecular viscosity”), which would affect larger proteins more than smaller ones (*30*, *31*).

The viscosity (η) of the cytoplasm of the cell is an elusive parameter to measure (*44*). The frictional force of such a complex medium cannot be captured in a single number because small molecules will experience (and impart) a different friction from large ones. Following the result that the observed diffusion coefficients scale with the complex molecular mass more markedly than predicted by the Einstein-Stokes equation, we hypothesize that the cytoplasm of *E. coli* is a non-Newtonian, dilatant fluid. A characteristic of dilatant fluids is that the viscosity increases with the stress applied to the fluid. Larger components inside the cell impose a higher pressure to the environment, which, in response, acts as being more viscous. We therefore argue that the viscosity of the cytoplasm should be considered as a function of the analyzed macromolecule, which will be subjected to a perceived viscosity depending on its size. We propose a new, revised version of the Einstein-Stokes equation (Eq. 3)

$$D=\frac{k_{B}T}{6\pi \eta_{\text{MW}}r}$$ (3)

where η_MW_ represents the perceived viscosity as a function of the molecular weight. Given the discrepancy between the observed diffusion coefficients and the values predicted by the original Einstein-Stokes equation, we propose a simple relationship between intracellular viscosity and complex molecular weight, which takes the form η = α*M*_complex_^0.20^ (Fig. 5E). By calculating the viscosity based on the observed diffusion coefficient, we propose that the perceived macromolecular viscosity varies from 9.02 to 15.02 centipoise (cP) for molecules ranging from 25.7 to 318.9 kDa. We believe that the relationship will not hold for metabolites or for megadalton macromolecules, as the polydisperse cytoplasm behaves like a fluid for small molecules, while it has glass-like properties for very big complexes (*15*).

### Possibility of static structures and damaged proteins at the cell poles

**Fig. 6.**
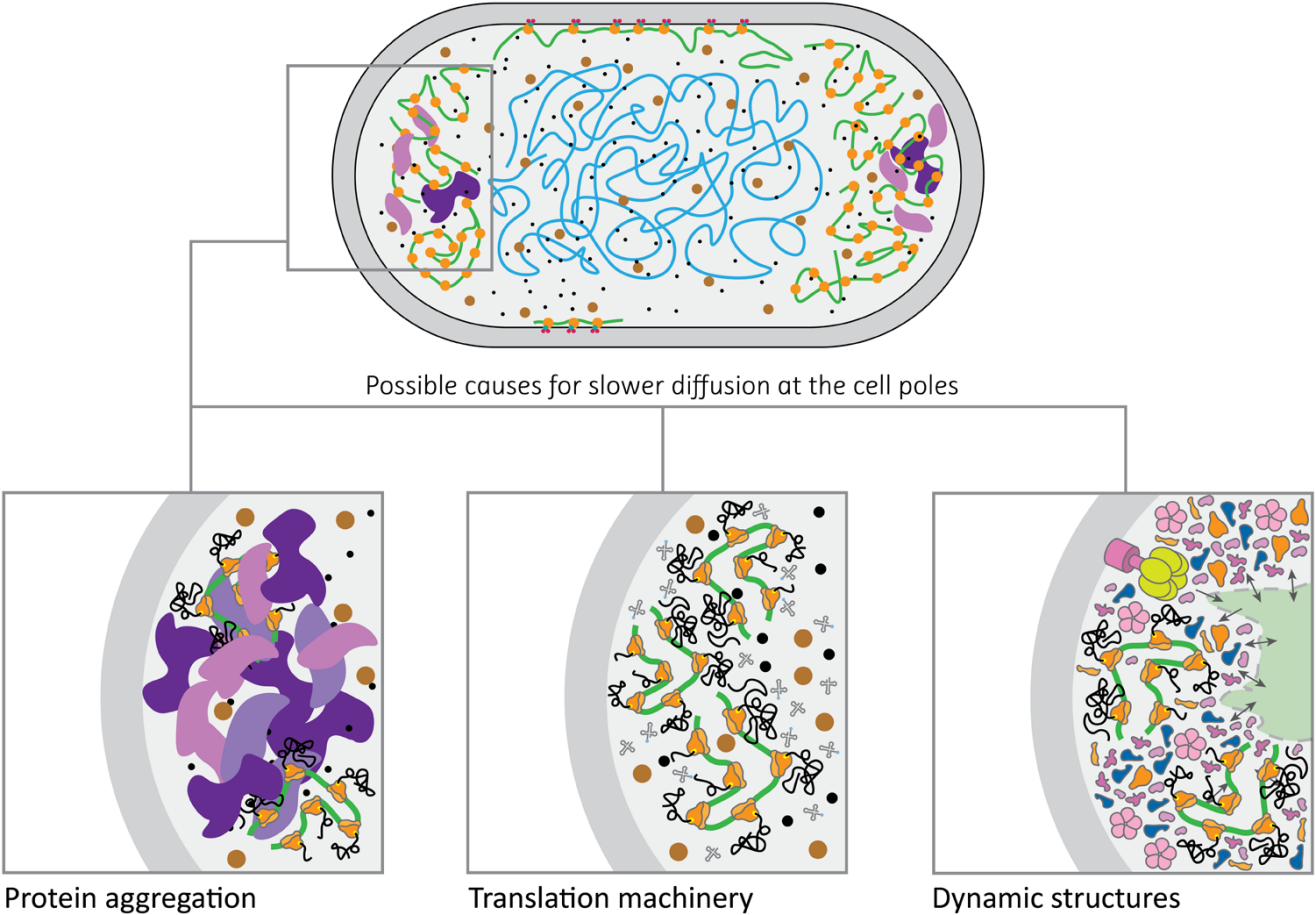
Possible scenarios for slower diffusion at cell poles. (**Left**) Protein aggregates (in purple) create a highly crowded environment. (**Middle**) Polysomes and transfer RNA molecules (in gray) form dynamic structures that slow down the diffusion of proteins. (**Right**) The hypothesis of a structured cytoplasm is depicted, where highly crowded regions would be responsible for the slower diffusion of proteins.

Comparison of the apparent diffusion coefficients shows that the in vivo mobility of the target proteins is 30 to 40% slower at the cell poles than in the cell center. This relative difference is substantially higher than the 5 to 10% slowdown observed at the poles of simulated *E. coli*–sized compartments with similar geometry (Fig. 5, C and D). While some of this disparity could be accounted for by the mismatch in the shape of the live cells and simulated compartments (i.e., live-cell poles are not perfect hemispheres), it is improbable that the minuscule differences in confining geometry would cause such a drastic difference in apparent diffusion coefficient. We consider such slowdown to be physiologically relevant, and we propose three possible explanations for this observation (Fig. 6): (i) accumulation of damaged proteins, where aggregated misfolded proteins are excluded from the nucleoid (*6*, *7*) and accumulate at the cell poles, giving rise to large, relatively immobile obstacles. These obstacles would have crowding and confining effects on proteins diffusing through the pole regions of the cell, decreasing their apparent diffusion coefficient; (ii) the translation machinery, which is known to be preferentially located in the cell poles, excluded from the nucleoid (*11*, *12*); and (iii) the existence of dynamic cytoplasmic structures situated at cell poles. A combination of these scenarios could also be possible. In all cases, the target proteins would experience a more crowded environment at the cell poles, explaining the slowdown of protein diffusion.

### Concluding remarks

We extended the recently developed technique SMdM to construct diffusivity maps of the *E. coli* cytoplasm at the scale of hundreds to tens of nanometers, and we determined the lateral diffusion coefficients of proteins in specific regions inside exponentially growing cells. We observe that protein diffusion solely depends on the mass of the protein complexes with no apparent effect of protein interactions. We provide a rationale for the deviation of the diffusion coefficients from the Einstein-Stokes equation and propose that the cytoplasm is a dilatant, non-Newtonian fluid. We also find that the lateral diffusion of the selected proteins is location dependent, with the cell poles displaying slower diffusion throughout the whole set of investigated proteins. The extent of the slowdown in the pole regions exceeds the confining effects of the cell membrane boundary, as inferred from computer simulations. We propose that this slowdown in diffusion is, in part, a consequence of macromolecular hindrance, be it from the accumulation of damaged proteins, localization of the translation machinery, or presence of dynamic cytoplasmic structures at the cell poles.

### MATERIALS AND METHODS

#### Databases

We searched the IntAct database (*34*) for all interactions annotated with the *E. coli* K12 taxonomy ID (8333) (*45*). Dataset download dates were 4 September 2018 and 8 December 2020. The abundance dataset was taken from Schmidt *et al.* (*3*). We selected the columns from the supplementary table with name, UniProt ID, functional annotation, and the abundance data for the cells grown in M9-glycerol media. The code and the data are available at https://github.com/MembraneEnzymology/smdm (*46*).

#### Target selection

We carried out the analysis of the IntAct database twice. Dataset accessed on 4 September 2018 was processed for the initial selection of target proteins for cloning and experimentation. Dataset accessed on 8 December 2020 was used to generate the tables and figures present in this publication to get the most recent context of the study.

#### Interactome dataset

The European Molecular Biology Laboratory European Bioinformatics Institute (EMBL-EBI) IntAct (*34*) database was accessed via website (www.ebi.ac.uk/intact/search), and *E. coli* K12 interactome was obtained by searching the term “taxid:83333” and was downloaded in the PSI-MI TAB 2.7 format (*47*). The search resulted in 28,943 and 29,417 binary interactions on 4 September 2018 and 8 December 2020, respectively.

#### Combining the datasets

The interactome and the abundance datasets were combined in a SQL (structured query language) database. The code that we used to construct and search the combined dataset is available at https://github.com/MembraneEnzymology/smdm/tree/main/Bioinformatic%20analyses (*46*). Briefly, we used PostgreSQL (www.postgresql.org) locally as the database system. We used psycopg2 (www.psycopg.org) as the adapter between Python and the PostgreSQL server. Within a PostgreSQL database, we created a table out of the IntAct (*34*) search results downloaded in the PSI-MI TAB 2.7 format (*47*). We then merged the table with the abundance dataset from Schmidt *et al.* (*3*), using the UniProt IDs as the common column. The best representation of the merged dataset is table S1, where each row corresponds to a binary interaction from IntAct. In addition, the table contains protein abundance data for each of the interactors, if the interactor is present in the abundance dataset. We then selected the UniProt IDs of proteins with copy number per cell of more than 1000, which resulted in 573 proteins of the 2359 total in the dataset, covering 93.6% of all proteins in the cell.

The combined dataset was used to query all 573 *E. coli* proteins with abundance above 1000 copies per cell. The following query conditions were used: (i) Protein UniProt ID matched with UniProt ID of either the interactor A or interactor B, (ii) the entry was specified as a physical interaction (MI:0914 or MI:0915), and (iii) taxonomy ID of at least one interactor had to be taxid:83333.

For each nonempty query result, we then created a table containing information for the searched UniProt ID. Among others, the columns include the following: (i) the number of all interactions found, (ii) whether the protein interacts with itself, (iii) whether the protein is annotated with the “cytoplasm” or “cytosol” Gene Ontology (GO) annotations (*48*, *49*) (go:"GO:0005737" and go:"GO:0005829", respectively), (iv) whether the protein associates with the large cellular components (cell membrane, DNA, RNA, and ribosomes), (v) whether the protein is annotated to be situated in the periplasm, (vi) whether the protein has a Protein Data Bank entry, (vii) the sum of the abundances of all of the protein’s interactors, and (viii) calculated loneliness (abundance in copies per cell divided by the sum of the abundances of all of the protein’s interactors). Note that all the information was taken from the two downloaded databases [IntAct (*34*) and Schmidt *et al.* (*3*)]. Filtering the information on GO annotations (*48*, *49*) other than the cytoplasm or cytosol was done using string matching, not by searching particular GO annotations. Those data were later evaluated manually using UniProt (*45*).

All query results were then combined into a master table containing 546 rows of search results for each queried UniProt ID that returned at least one interaction; 27 proteins returned no interactions. All the entries in the master table were then divided into deciles with regard to their “molecular weight,” “abundance,” and “loneliness” columns.

From the master table, we manually chose around 50 initial targets, prioritizing cytoplasmic proteins, their lack of interactions with the large cellular components, and their spread among the overall population in terms of molecular weight, abundance, and loneliness. All of those were then verified manually using the UniProt database (*45*). In the end, we settled on 18 proteins (table S1).

We based our final target selection on: (i) protein being cytoplasmic; (ii) protein not having known interactions with the large cellular components; (iii) ranking with regard to loneliness, molecular weight, and abundance; (iv) availability of the C terminus for tagging; and (v) oligomeric state, which, in combination with the molecular weight, yielded the mass of the protein complex. Points 1 to 3 were addressed automatically, and the most promising targets were then searched manually in UniProt (*45*) to address points 4 and 5, yielding a set of 18 native *E. coli* proteins for subsequent experimental work. In addition, the thioredoxin genes from *L. lactis* and *H. volcanii* were searched in UniProt (*45*), and *trxA2* from *H. volcanii* and trxA from *L. lactis* were included as non-native proteins that are homologs of TrxA from *E. coli*.

#### Strains and genes

We used *E. coli* BW25113 [F-, Δ*(araD-araB)567,* Δ*lacZ4787(::rrnB-3),* λ*-, rph-1,* Δ*(rhaD-rhaB)568, hsdR514*], unless stated otherwise. In addition, for cloning and storage of intermediate constructs, we used *E. coli* DH5α [F-, Δ*(argF-lac)169,* φ*80dlacZ58(M15),* Δ*phoA8, glnX44(AS),* λ*-, deoR481, rfbC1, gyrA96(NalR), recA1, endA1, thiE1, hsdR17*] and MC1061 [F-, Δ*(araA-leu)7697, [araD139]_B/r_,* Δ*(codB-lacI)3, galK16, galE15(GalS),* λ*-, e14-, mcrA0, relA1, rpsL150(strR), spoT1, spoT1, hsdR2*]. All native *E. coli* genes were obtained by polymerase chain reaction (PCR) cloning from the BW25113 chromosome using specific primers. *L. lactis* NZ9000 was used as a source of *trxA*_Lla_; *trxa2_Hfxv_* and *mEos3.2* genes were obtained by purchasing *E. coli*–optimized nucleotide sequences (GeneArt Service, Thermo Fisher Scientific). We chose mEOS3.2 (*32*) as the fluorescent tag used for SMdM. All proteins were tagged on their C terminus, and the mEOS3.2 protein sequence was preceded by a -Gly-Gly-Tyr-Gly-Gly-Ser- linker, with the N-terminal methionine of mEOS3.2 substituted for glycine.

#### Gene cloning

Each amplified gene was ligated into a pBAD vector carrying the mEos3.2 gene, using the USER cloning protocol (*50*). Briefly, we designed a general pair of primers to amplify the pBAD-mEos3.2 vector, which we used as a backbone for every construct. We then designed a specific pair of primers for every gene (Table 2), so that they had a 5′ region overlapping for 8 to 12 nucleotides with the 5′ region of the primers used to amplify the vector. All primers contained a single deoxyuracil residue flanking the 3′ end of the complementary region. All PCR products were treated with the restriction enzyme Dpn I for 1 to 2 hours at 37°C to remove any trace of methylated DNA. All the DNA fragments were then purified using the NucleoSpin Gel and PCR clean-up kit (MACHEREY-NAGEL). Purified fragments were mixed together in a 1:3 vector-to-gene molar ratio, using 100 ng of the vector-DNA and the proper amount of the gene-DNA. USER enzyme (1 μl; New England BioLabs) was added to the DNA mix, together with the appropriate volume of the Cutsmart (New England BioLabs) reaction buffer. The final reaction volume was reached by filling with sterile Milli-Q to 10 μl. The reaction was incubated between 30 and 60 min at 37°C, followed by a further incubation period between 30 and 60 min at room temperature. Five microliters of the reaction was then used to transform 100 μl of chemically competent *E. coli* MC1061. DNA was then isolated via plasmid preparation, using the NucleoSpin Plasmid kit (MACHEREY-NAGEL), and subsequently sequenced via Sanger sequencing by Eurofins Genomics.

**Table 2.** List of all primers used to perform the cloning. Columns represent the following, from left to right: type of fragment (whether it is the backbone used as vector or an insert), name of the vector or of the gene of interest, source of the DNA (whether it is a plasmid or the chromosome), origin (whether it was ordered as a synthetic gene or extracted from the chromosome), direction of the primer, and sequence in 5′ to 3′ direction. The uracil is highlighted in bold. Fw, forward; Rv, reverse.

**Cloning fragment**	**DNA name**	**DNA type**	**DNA origin**	**Primer**	**Primer sequence (5′ → 3′)**
Backbone	pBAD-mEos3.2	Plasmid	GeneArt	Fw	AACTGGTGGA**U**CCGGTAGC
				Rv	ATGGTTAAT**U**CCTCCTGTTAGCCC
Insert	*aceA*	Chromosome	BW25113	Fw	AATTAACCA**U**GAAAACCCGTACACAACAAATTGAAG
				Rv	ATCCACCAGT**U**CCACCAAACTGCGATTCTTCAGTGGAGC
Insert	*thrC*	Chromosome	BW25113	Fw	AATTAACCA**U**GAAACTCTACAATCTGAAAGATCACAACGAG
				Rv	ATCCACCAGT**U**CCACCCTGATGATTCATCATCAATTTACGCAACG
Insert	*nadE*	Chromosome	BW25113	Fw	AATTAACCA**U**GACATTGCAACAACAAATAATAAAGGC
				Rv	ATCCACCAGT**U**CCACCCTTTTTCCAGAAATCATCGAAAACGG
Insert	*sucC*	Chromosome	BW25113	Fw	AATTAACCA**U**GAACTTACATGAATATCAGGCAAAACAAC
				Rv	ATCCACCAGT**U**CCACCTTTCCCCTCCACTGCGGC
Insert	*slyD*	Chromosome	BW25113	Fw	AATTAACCA**U**GAAAGTAGCAAAAGACCTGGTGG
				Rv	ATCCACCAGT**U**CCACCGTGGCAACCGCAACCG
Insert	*osmC*	Chromosome	BW25113	Fw	AATTAACCA**U**GACAATCCATAAGAAAGGTCAGGC
				Rv	ATCCACCAGT**U**CCACCCGATTTCAACTGATAATCCAGCGTAATTTCCG
Insert	*grxC*	Chromosome	BW25113	Fw	AATTAACCA**U**GGCCAATGTTGAAATCTATACC
				Rv	ATCCACCAGT**U**CCACCTTTCAGCAGGGGATCC
Insert	*ilvC*	Chromosome	BW25113	Fw	AATTAACCA**U**GGCTAACTACTTCAATACACTG
				Rv	ATCCACCAGT**U**CCACCACCCGCAACAGC
Insert	*aceB*	Chromosome	BW25113	Fw	AATTAACCA**U**GACTGAACAGGCAACAACAACC
				Rv	ATCCACCAGT**U**CCACCCGCTAACAGGCGGTAGCC
Insert	*acpP*	Chromosome	BW25113	Fw	AATTAACCA**U**GAGCACTATCGAAGAACGCG
				Rv	ATCCACCAGT**U**CCACCCGCCTGGTGGCCGTT
Insert	*erpA*	Chromosome	*E. coli* BW25113	Fw	AATTAACCA**U**GAGTGATGACGTAGCACTGC
				Rv	ATCCACCAGT**U**CCACCGATACTAAAGGAAGAACCGCAACCG
Insert	*ndk*	Chromosome	BW25113	Fw	AATTAACCA**U**GGCTATTGAACGTACTTTTTCCATC
				Rv	ATCCACCAGT**U**CCACCACGGGTGCGCGGG
Insert	*trxA*	Chromosome	BW25113	Fw	AATTAACCA**U**GAGCGATAAAATTATTCACCTGACTGAC
				Rv	ATCCACCAGT**U**CCACCCGCCAGGTTAGCGTCGAG
Insert	*trxA_Lla*	Chromosome	*L. lactis* MG1363	Fw	AATTAACCA**U**GGAATATAATATTACTGATGCAACGTTTGATAAAG
				Rv	ATCCACCAGT**U**CCACCTGATAATTCAGCAATCACGGCTTTAAG
Insert	*trxA_Hvo*	Plasmid	GeneArt	Fw	AATTAACCA**U**GAGCACCCCGAAAACC
				Rv	ATCCACCAGT**U**CCACCTGCTGCTGCTTCAATAATATCG
Insert	*leuS*	Chromosome	BW25113	Fw	AATTAACCA**U**GCAAGAGCAATACCGCC
				Rv	ATCCACCAGT**U**CCACCGCCAACGACCAGATTGAGGAG
Insert	*leuB*	Chromosome	BW25113	Fw	AATTAACCA**U**GTCGAAGAATTACCATATTGCCG
				Rv	ATCCACCAGT**U**CCACCCACCCCTTCTGCTACATAGCG
Insert	*metK*	Chromosome	BW25113	Fw	AATTAACCA**U**GGCAAAACACCTTTTTACGTCC
				Rv	ATCCACCAGT**U**CCACCCTTCAGACCGGCAGCATCG
Insert	*icd*	Chromosome	BW25113	Fw	AATTAACCA**U**GGAAAGTAAAGTAGTTGTTCCGG
				Rv	ATCCACCAGT**U**CCACCCATGTTTTCGATGATCGCGTCAC

Chemical competent cells were prepared according to protocol (*51*). *E. coli* MC1061 cells were transformed with the final product of USER cloning. *E. coli* BW25113 cells were transformed with DNA obtained via plasmid preparation, performed using the NucleoSpin Plasmid kit (MACHEREY-NAGEL). Transformation was performed with the heat shock method (*51*).

#### Media for cell culturing

Lysogeny broth (LB) (*52*) was prepared following the formula of 10/10/5% (w/v) in MilliQ of NaCl, tryptone (Formedium), and peptone (Formedium), respectively. The medium was sterilized by autoclaving. Mops-buffered minimal medium (MBM) was prepared following the formula in (*53*). Briefly, we prepared the macro- and micronutrient solutions and mixed them to obtain concentrated base MBM, which was adjusted to pH 7.4 with 2 M KOH. We then added MilliQ to obtain a 10× concentration of the final medium. The 10× base MBM was then sterilized by filtration using 0.2-μm filters (Cytiva), aliquoted into 50-ml tubes, and stored at −20°C.

Final MBM used for cell growth was prepared as follows: 50 ml of 10× base MBM was thawed and diluted to approximately ~5×. We then added 5 ml of 132 mM K_2_HPO_4_ plus 7.28 ml of 4 M NaCl, both filter-sterilized. The NaCl was added to reach the desired final osmolality of approximately 0.28 osmol/kg, and the volume added was determined with a calibration curve. The solution was then filled to 500 ml with autoclaved MilliQ, giving 1× MBM. The medium in this form was stored at 4°C for up to 2 months. Right before culturing, MBM was supplemented with sterile glycerol and ampicillin to final concentrations of 0.2% (v/v) and 100 μg/ml, respectively. This MBM, supplemented with carbon source and antibiotic, gave pH values in the range of 7.22 to 7.26 and osmolality in the range of 0.28 to 0.30 osmol/kg, as measured by a cryoscopic osmometer (Gonotec Osmomat 3000). We prewarmed the medium to the temperature of growth, typically 30°C.

#### Preculturing of cells for microscopy

##### Day 1—LB preculture

For each experiment, we took a glycerol stock of *E. coli* bearing one of the constructs reported in table S1, and we scratched it with a sterile inoculation loop. We then dipped the loop in 3 ml of LB medium containing ampicillin (100 μg/ml). We incubated the preculture overnight at 30°C, with shaking at 200 rpm at a 45° angle; for all culturing, we used 14-ml plastic culturing tubes (Greiner Bio-One). After the incubation, the cultures were in the stationary phase and had an optical density at 600 nm (OD_600_) above 2.

##### Day 2—MBM preculture

On day 2, we transferred 30 μl of the LB preculture to 3 ml of MBM containing 0.2% (v/v) glycerol and ampicillin (100 μg/ml). The culture was incubated overnight at 30°C, with shaking at 200 rpm. After the incubation, the culture OD_600_ was in the range of 0.8 to 1.8.

##### Day 3—MBM culture and preparation for the measurement

The next day, approximately 4 to 6 hours before microscopy, the overnight MBM precultures were diluted in fresh, prewarmed MBM with 0.2% (v/v) glycerol plus ampicillin (100 μg/ml) to a final OD_600_ of 0.1 to 0.2. The diluted cultures containing pBAD-mEos3.2 were induced with 0.1% (w/v) l-arabinose at the moment of dilution to achieve an expression level high enough for microscopy measurements. The diluted cultures of fusion constructs were then incubated at 30°C, with shaking at 200 rpm until the measurement, and induced with 0.1% (w/v) l-arabinose approximately 1 to 2 hours before microscopy.

The diffusion measurements were performed on cultures that (i) had at least doubled their OD since dilution, (ii) did not exceed OD_600_ of 0.6, and (iii) were induced with l-arabinose for at least 1 hour. Effectively, the culture OD_600_ at measurement was in the range of 0.22 to 0.57. From the final OD_600_ measurement onward, the cells were handled at room temperature. Right before the measurement, the cultures were spun down in a tabletop centrifuge and concentrated three to six times in the growth medium. The concentrated cell suspension was immediately used for microscopy.

#### Live-cell single-molecule microscopy

##### Preparation of glass slides

High-precision glass slides [24 mm by 60 mm, 170-μm (1.5H) thickness (Carl Roth GmbH & Co KG)] were sonicated in 5 M KOH for 45 min and then rinsed 10 times with MilliQ. The remaining MilliQ was removed with pressurized air, and dry slides were stored and protected from dust for up to 5 days.

##### Preparation of agarose pads

Agarose (Duchefa Biochemie) was added to MilliQ to the final concentration of 1.5% (w/v) and autoclaved. Afterward, it was stored at room temperature for up to a month. MBM (2×) was prepared similarly to 1× MBM, with the final addition of sterile MilliQ up to 250 instead of 500 ml (from 50 ml of 10× base MBM). Polydimethylsiloxane (PDMS) chambers for the agarose pads were prepared by cutting out approximately 20 mm by 20 mm by 4 mm of flat PDMS chunks. A circular opening for the agar was made by punching through the PDMS chunk with an 8-mm-diameter biopsy needle. The PDMS chambers are reusable, with cleaning by sonication in 70% ethanol for 45 min and drying with compressed air.

Approximately half an hour before the measurement, the 1.5% agarose gel was reheated in a microwave oven until it melted completely. In the meantime, 4 ml of 2× MBM was supplemented with glycerol to the final concentration of 0.4% (v/v), and the medium was preheated in a 50°C incubator. Up to three clean PDMS chambers were placed tightly spaced on a clean glass slide. The chambers were gently pressed against the glass slide to ensure proper sealing. Four milliliters of preheated 2× MBM supplemented with 0.4% (v/v) glycerol was mixed with 4 ml of melted 1.5% agarose and mixed gently. The solution was then quickly poured into the PDMS chambers until they filled. The agar was left to solidify in the open air inside a laminar flow hood to ensure sterility. After the agar solidified, the chambers with the pads inside were gently covered with a glass slide to prevent evaporation. The chambers are used within 7 hours of preparation. The pads made in this way display no notable background increase during single-molecule microscopy measurements, as compared to fresh MBM.

##### Live-cell imaging

To ensure a constant temperature of the microscope throughout the imaging process, we turned on the equipment 4 to 5 hours before the measurement. This allowed us to minimize the *xy* drift of the samples, which would otherwise have been more pronounced because of fluctuations in stage and ambient temperatures. A 561-nm laser (Coherent, OBIS) beam was focused in the center of the camera detector, such that the circular beam had a diameter slightly smaller than one side of the detector.

The cells were imaged on a clean, nonfunctionalized glass. Immobilization was achieved by depositing 5 μl of the concentrated cell suspension in the center of the glass slide and then pressing the cells against the glass surface with solidified agarose pads, formed inside a PDMS chamber. The agarose pad had a final concentration of agarose at 0.75% (w/v), as well as 1× MBM and 0.2% (v/v) glycerol (see preparation of agarose pads). Therefore, apart from the ampicillin, the agarose pad had medium parity with the liquid MBM. The top of the PDMS/agarose chamber was covered with a 22 mm by 22 mm glass slide to prevent evaporation. Usually, the immobilization process results in some spillover of the cell suspension from beneath the PDMS chamber. That is blotted away with a paper tissue, because we observed that spillover extended the time needed for the cells to settle. We then proceed to image the cells trapped below the agarose pad in wide-field mode. Immediately after placing the chamber on top of the cell suspension droplet, the cells remained mobile. It usually took 2 to 10 min for the cells to settle completely. Once immobilized, the cells did not move for the duration of the experiment. We did not observe any drying of the sample or cell growth throughout the 35 to 60 min of the measurement.

After the cells went still, we picked a field of view where cells were in close proximity to one another but not too densely packed. Low density of cells would require us to use large selections for acquisition, increasing the total measurement time. Cells being too close to one another creates a problem later in the analysis, where pairing of foci from different cells potentially leads to artifacts. We preferred fields of view where cells were distanced at least 1 μm away from each other, but there were at least five cells in a 200 × 200 pixel area. We positioned the most promising area in the center of the detector to ensure the best illumination. We then created a rectangular selection encompassing the cells that we chose. The minimal time of exposure of the detector scales with the number of pixels within the acquired selection, which translates directly to the total time of the measurement. Therefore, we sought to minimize the size of the acquired selection. The selections were within the range of 140 pixels (14 μm) to 256 pixels (25.6 μm) on each axis. After the selection was made, we switched to the shortest possible acquisition time and focused the sample in the middle of the cells. We then enabled the autofocus function of the microscope to avoid *z* drift. We then switched to the dark-field mode of the microscope, with low laser powers and continuous illumination at high camera gain. Focus was further adjusted to produce the brightest and smallest foci. We adjusted the laser beam angle to give us the highest number of foci, which resulted in the beam angle slightly below than the critical angle for total internal reflection [highly inclined and laminated optical sheet microscopy (*54*)]. After adjusting the focus and the beam angle, the camera and the lasers were synchronized in the stroboscopy mode: First, we determined the minimal amount of time necessary to illuminate mEos3.2 in *E. coli* to obtain reasonable foci. We established that using our setup, the illumination of 0.5 ms of duration at 135 mW of 561-nm laser output produced detectable foci. Second, we verified the camera dead time to be at 0.78 ms with an oscilloscope (Hameg Instruments, 100-MHz analog scope, HM1004), which followed the camera specification.

Using a custom Python notebook, with the use of the nidaqmx package (https://github.com/ni/nidaqmx-python), we synchronized the illumination to the electrical pulses generated by the camera at the beginning of the dead time. The acquisition time (exposure time + dead time) is read out automatically using a programmable card (National Instruments, PCI – 6602) and was used to calculate the time needed to position laser pulses such that the frames can be paired according to the schematic presented in Fig. 3. For a detailed overview of the scripts used for managing the microscope, please refer to https://github.com/MembraneEnzymology/smdm/tree/main/Microscopy (*46*).

The camera pulse of the first frame in the pair generates countdowns to the four following events: (1) ignoring the following camera pulse, (2) start of the activator 405-nm laser pulse, (3) start of the first excitatory 561-nm laser pulse, and (4) start of the second excitatory 561-nm pulse. Countdown 1 determines the total time of the pulse sequence. This is equal to the acquisition time with an added arbitrary time interval. In this way, a single camera pulse following the initial one is ignored, ensuring the every-other-frame stroboscopic illumination. Countdown 2 determines the start of the 405-nm laser pulse, meant to photoswitch the mEOS molecules. The 405-nm laser (Coherent, OBIS) pulse duration was 1 ms. We chose this interval and pulse length to ensure that the 405-nm pulse was confined to the first frame of the illumination. Countdowns 3 and 4 determine the time of the two excitatory 561-nm laser pulses.

Throughout the measurements, we are adjusting the laser intensity of the 405-nm pulse, gradually increasing it as we were observing the number of fluorescent spots diminishing over time. We typically acquire three to six consecutive movies per field of view, totaling 100,000 to 160,000 frames. The total measurement time, from centrifugation to the end of acquisition, is between 35 and 60 min. The total number of frames and length of the measurement depend on (i) frame acquisition time (pixel size of the measured area), (ii) density of the immobilized cells, and (iii) time the cells need to settle below the agar pad. During all of the measurements, the stage temperature was at 21° ± 1°C, and the ambient temperature was at 20° ± 0.5°C.

#### SMDM analysis

##### Single-molecule displacement analysis

For each field of view, we recorded several consecutive movies to allow for a faster storage of the files. To preserve the correct order of excitation throughout the whole movie, before concatenation, we analyzed each movie for intensity of odd and even frames. Because of the limitations of the 561-nm laser, the second frame in the excitation order had lower background intensity. We use the background intensity data to align individual movies, by deleting or keeping the first frame of each movie. This results in one large .stk file for each field of view. For details, see functions determine_first_image and concatenate_movies in Concatenate_and_find_peaks.ipynb available at https://github.com/MembraneEnzymology/smdm/tree/main/Microscopy (*46*).

#### Single-molecule detection

For single-molecule analysis, we used the Storm-analysis package developed by the Zhuang laboratory (http://zhuang.harvard.edu/software.html). We used the 3D-DAOSTORM program for peak detection (*55*). We tune the detection parameters to efficiently pair digital peaks with the observed foci. The movie is split into several parts that are analyzed simultaneously. After the full movie was analyzed, the localizations were corrected for *xy* drift. For parameters and more detail, see functions find_all_peaks and analyze in Concatenate_and_find_peaks.ipynb available at https://github.com/MembraneEnzymology/smdm/tree/main/Microscopy (*46*).

#### Peak pairing

To obtain displacements from the movies, we paired the localizations from the two consecutive frames of the stroboscopic illumination pattern. We set the maximum distance between any two peaks to be paired, which is dependent on the diffusion coefficient and was adjusted per field of view; for all our constructs, we set the distance to 600 nm. This distance is then used to find all possible peak pairs for each couple of frames. If the peaks are too close to one another, then it creates an ambiguity in the assignment of peak pairs. We correct for this ambiguity by applying a linear background term (see the “Data fitting” section). To obtain a displacement, we match each peak in the first frame of the couple with all the peaks falling within the maximum chosen distance (600 nm) in the second frame. Displacements are then binned inside a region (either a cell area or a pixel) on the basis of their starting position, that is, their position in the first frame of the couple. This procedure is repeated for all frame couples of each field of view. For details, see Analysis_script.ipynb available at https://github.com/MembraneEnzymology/smdm/tree/main/Microscopy (*46*).

#### Cell detection and rotation

Cells were detected automatically using the Voronoi clustering method (*56*) integrated in the Python library SciPy (https://docs.scipy.org/doc/scipy/reference) (*57*). For each cluster constituting cells, eigenvectors are obtained through the calculation of the covariance matrix. The angle between the first eigenvector and the *x* axis is then calculated. Subsequently, an appropriate rotational matrix is applied to the *xy* coordinates to align the major axis parallel to the *x* axis of the diffusion map (see code for more details). This yields a better resolution (number of peak pairs) per pixel, owing to the squared shape of the pixels. Each cell is then extracted from the field of view and analyzed in its own frame of reference, in which the center of the cell is placed at the origin of an *x*-*y* system of coordinates. All cells were inspected to ensure that they did not present signs of aggregations or errors due to the clustering method.

Cells were then filtered on the basis of their number of displacements. We calculated the median of the number of displacements per cell for each analyzed protein, and we used the minimum and the maximum of these medians, 2000 and 20,000, respectively, to define a range of displacements. We then analyzed only those cells that had a number of displacements within such range. This was done to minimize the error in determining the diffusion coefficient caused by having a too little number or a too large number of displacement (fig. S6).

A pixel map with pixel size between 50 and 200 nm was obtained from each cell. Every pixel contains the information of all the peak pairs (hence of all the displacements) for which the starting position is located inside the pixel. This map is then fitted (see the Data fitting section). If the number of displacement per pixel is not high enough to pass the minimal threshold for fitting (set to 10), then the map is incomplete, and the pixel size is increased.

#### Cell area selection

Cell area selections were made from vertical lines along the cell long axis (Fig. 4A). The leftmost displacement and the rightmost displacement were used to calculate the cell length. Twenty percent of the total length was added to the leftmost *x* coordinate and subtracted from the rightmost *x* coordinate to create the pole regions and the cell center. We used 20% of the total length to identify each pole; the average cell dimensions were 2.25 μm for the length and 0.45 μm for the radius (fig. S2). By calculating the ratio between radius and length, one obtains 0.45/2.25 = 0.2; hence, the radius represents 20% of the total length. Because the radius is the parameter that determines the size of the hemisphere constituting the pole, we chose 20% of the length as an estimate of the pole area. We incorporated in our analysis the totality of displacements present in the cell.

We then applied a filter to exclude from the analyses those cells for which the displacements in a specific area were less than 100, to have a maximum error of 10% in determining the diffusion coefficient (*33*). In this case, the cell center *D*_app_ value includes displacements close to the cell membrane, but here, the outcome is less biased by the density of the data. Each area was then fit using the PDF described in Eq. 1 (Fig. 4B).

#### Data fitting

Fitting of the data was performed using an adjusted two-dimensional PDF for displacements. The normal two-dimensional PDF for displacements is described by the Rayleigh distribution

$$p(r,t)=\frac{2r}{4\mathrm{Dt}}e^{-\frac{r^{2}}{4\mathrm{Dt}}}$$ (4)

We introduced a linear background term to take misassigned displacements into account, that is, in cases where multiple particles are detected in the same frames, and the wrong starting positions and final positions are paired with each other. The equation with linear background term has the form

$$p(r,t)=\frac{2r}{4\mathrm{Dt}}e^{-\frac{r^{2}}{4\mathrm{Dt}}}+\mathrm{kr}$$ (5)

This equation, however, does not represent a PDF anymore, because its integral is not 1. Hence, we applied a correction factor to Eq. 8, which led to

$$\begin{array}{cc} p(r,t) & =\frac{1}{\int_{0}^{r_{\text{max}}}p(r,t)\mathrm{dr}}(\frac{2r}{4\mathrm{Dt}}e^{-\frac{r^{2}}{4\mathrm{Dt}}}+\mathrm{kr})\\ & =\frac{1}{1-e^{-\frac{r_{\text{max}}^{2}}{4\mathrm{Dt}}}+\frac{k}{2}r_{\text{max}}^{2}}(\frac{2r}{4\mathrm{Dt}}e^{-\frac{r^{2}}{4\mathrm{Dt}}}+\mathrm{kr}) \end{array}$$ (6)

Because the integral was evaluated from 0 to *r*_max_ (600 nm in our case), we filtered the acquired data such that displacements larger than 600 nm were excluded from our dataset. In the case of simulated data, where there is no background effect, we fit our data using Eq. 7. We fit the data to the model using maximum likelihood estimation (*58*).

We chose 600 nm as the value for *r*_max_ with the following reasoning: The root of the mean square displacement is given by

$$\text{RMSD}=\sqrt{2n\mathrm{Dt}}$$ (7)

where *n* is the number of dimensions. Rearranging Eq. 7, one can obtain

$$D=\frac{{\text{RMSD}}^{2}}{2\mathrm{nt}}$$ (8)

Considering *n = 2* (because we do not have information about the *z* coordinate of diffusing molecules with our setup), we obtain *D* = 60 μm^2^/s, which is a value about five times larger than the one reported for fluorescent proteins diffusing in the cytoplasm of *E. coli*. This gave us a confidence interval big enough to ensure the monitoring of all the displacements of all the analyzed proteins.

The diffusion coefficients obtained from the data points located in the central part of the cells were averaged for each protein construct. The average diffusion coefficient was then analyzed as a function of other parameters (abundance, loneliness, molecular weight, and complex mass). Because a trend was observed between diffusion and complex mass, we fitted the dependence of the diffusion coefficient on complex mass with a power law relationship *D =* α*M*_complex_^β^, where *M*_complex_ is the complex mass and α and β are fitting parameters. We performed the fitting using the function curve_fit included in the SciPy library (*57*), which allowed us to directly obtain both α and β, as well as the associated errors as the diagonal values of the covariance matrix.

#### Simulations

##### Smoldyn input and output

Simulations were conducted with the Smoldyn software (www.smoldyn.org) (*36*). The script used for Smoldyn simulations is provided at https://github.com/MembraneEnzymology/smdm/tree/main/Simulations (*46*). Smoldyn simulates the random walk of particles by calculating the mean square displacement for each particle based on the input diffusion coefficient and time steps. It then randomly selects a displacement from a normal distribution of displacements having as mean the squared mean square displacement. Furthermore, the program also randomly selects a direction in the three axes *x*, *y*, and *z*. This process is applied individually for every particle, meaning that each particle will have its unique direction and displacement for every time step.

We generated two distinct datasets: one for analyzing the differences between input diffusion coefficient and apparent diffusion coefficient and one for analyzing the differences between cell center and cell poles. For the first dataset, we kept the size of the simulated cell constant, and we generated 25 replicates for each simulated diffusion coefficient. Briefly, we generated a spherocylinder with a radius of 0.45 μm and a length of 2.25 μm. The surface of the spherocylinder is described as reflective, meaning that simulated particles bouncing against it are reflected without loss of kinetic energy. We then placed particles inside the spherocylinder at random starting positions, i.e., uniformly distributed. The particles are described as mathematical points, meaning that they do not interact with one another. For input diffusion coefficients greater than or equal to 1 μm^2^/s, we used 25 particles; for diffusion coefficients greater than or equal to 0.05 μm^2^/s and smaller than 1 μm^2^/s, we used 100 particles; and for the diffusion coefficient of 0.01 μm^2^/s, we used 500 particles. This was done to ensure a homogeneous distribution of particles throughout the entire simulation. We then simulated diffusion of the particles for a total of 2 s, with time steps of 0.1 ms. For the second dataset, we generated 346 cells varying in size and in input diffusion coefficient: Various combinations of cell diameters (ranging from 0.2 to 2.34 μm), cell lengths (ranging from 1.1 to 3.64 μm), and diffusion coefficients (ranging from 0.5 to 20 μm^2^/s) were used to simulate the motion of 25 particles randomly placed inside the spherocylinders. We again simulated the diffusion of the particles for a total of 2 s, with time steps of 0.1 ms.

The output of the simulation is presented in the form of a table in which the first column presents the time steps, and the following columns give the *x*, *y*, and *z* positions of each particle; the second, third, and fourth columns define the spatial position of the first particle; the fifth, sixth, and seventh columns define the position of the second particle; and so on. Each row then identifies the *xyz* coordinates of all particles for each time step.

##### Smoldyn output management

The output from Smoldyn simulation is managed in Python, using the Pandas (https://pandas.pydata.org) (*59*) and NumPy (https://numpy.org) (*60*) libraries. A detailed version of the code is given at https://github.com/MembraneEnzymology/smdm/tree/main/Simulations (*46*). Briefly, because the time step in the experimental setup is 1.5 ms, whereas Smoldyn uses 0.1 ms, we zipped together the data from each *i* row of the output table described in the previous section with the data from each *i* + 15 row. We then treat the particles in each zipped row as if they are displacements observed in a time step of 1.5 ms. This allowed us to effectively obtain 19,985 displacements per particle; the last 15 rows could not be paired with any other row, and so they were considered as final positions.

The displacements are then binned in space on the basis of their starting position. The area used for the binning of the displacements determined the pixel size of the generated map. Each pixel contained pairs of positions of particles that were virtually identical to what we obtained experimentally in the peak pairing process (see the “Peak pairing” section). The virtual cells are then analyzed in the same way as the experimental data in terms of cell area selection and the data fitting process.

#### TrxA analysis

Because published structures of wild-type TrxA were not available at the time of writing of this manuscript, we used a modeling tool to obtain the three-dimensional configuration of the protein. TrxA models were made using the SWISS-MODEL workspace (*61*, *62*). The amino acid sequences for *E. coli*, *L. lactis*, and *H. volcanii* TrxA were obtained from the UniProt database (*45*), using the accession numbers P0AA25, A0A089XQE8, and A0A558GDT3, respectively. For *E. coli* TrxA, the structure 3DXB.1.A (*63*) was used as a template, giving a monomeric model with a global model quality estimation (GMQE) of 0.88 and a qualitative model energy analysis (QMEAN) of 0.77. For *L. lactis* Trxa, the structure 2O87.1.A (*64*) was used as a template, giving a monomeric model with a GMQE of 0.74 and a QMEAN of 0.01. For *H. volcanii* TrxA, the structure 6KIL.1.A (*65*) was used as a template, giving a monomeric model with a GMQE of 0.74 and a QMEAN of −0.41. For details about GMQE and QMEAN, we redirect the reader to the help page from SWISS-MODEL (https://swissmodel.expasy.org/docs/help).

The models were downloaded in pdb format. Molecular graphics and analyses were performed with UCSF Chimera (*66*), developed by the Resource for Biocomputing, Visualization, and Informatics at the University of California, San Francisco, with support from NIH P41-GM103311. The software was used to automatically add hydrogen and charges to the proteins, using the default parameters. Afterward, Coulombic surface coloring was applied to visualize the surface charge distributions of the models. The dipole moment was calculated using the script dipole.py available in the scripts page of the Chimera wiki repository.

#### Statistical analyses

All statistical analyses were performed using the Python package stats from the SciPy library (*57*). Spearman’s rank correlation test (*67*) was used to check whether there was correlation between datasets, such as for the diffusion coefficients of the different proteins and their abundance, loneliness, molecular weight, complex mass, and their perceived viscosity. Spearman’s test returns a value between −1 and 1. The closer this value is to the extremities, the more the datasets will be negatively or positively correlated. In general, values above 0.6 or below −0.6 are considered as a result of a moderate correlation, and values above 0.8 or below −0.8 are considered a result of a strong correlation. The analysis of the residual was performed by plotting the observed value subtracted of the value predicted by the fit over the value predicted by the fit. Residuals higher than zero indicate an underestimation from the fit, while residuals lower than zero indicate an overestimation.

Shapiro-Wilk test for normality (*68*) was used to check whether the data were normally distributed. The test assumed the null hypothesis that the data were normally distributed, and we set a level of confidence of 1%. Hence, for *P* > 0.01, the null hypothesis could not be rejected, and the distribution was assumed to be normal. This analysis was necessary to perform the proper statistical analyses on the dataset. If data were not normally distributed, then a Mann-Whitney *U* rank test (*69*) was performed to check for significant differences between datasets.

In the case of non-normally distributed datasets, the distributions were visually analyzed via kernel density estimation (*70*). Briefly, a kernel is obtained by the data underlying the distribution, and for each data point in the dataset, a Gaussian function centered in that point is described. The sum of all the Gaussians provides a continuous distribution that describes the underlying data.

The Mann-Whitney *U* rank test was used to determine whether the means of two non-normally distributed populations are equal, such as for the analyses performed on the cell poles. Results are considered significant for *P* < 0.01.

The *F* test was used to compare constrained and unconstrained fitting of the power law equation (see the “On cytoplasmic viscosity” section in Supplementary Text). Results are considered significant for *P* < 0.05.

## Supplementary Materials

### This PDF file includes:

Supplementary Text Figs. S1 to S6 Table S1 References

## Notes

View/request a protocol for this paper from *Bio-protocol*.
