# Supplementary material for: Protein diffusion in Escherichia coli cytoplasm scales with the mass of the complexes and is location dependent
Source: Sci Adv. 2022 Aug 12;8(31):eabo5387. doi: 10.1126/sciadv.abo5387 (PMC9374337; doi:10.1126/sciadv.abo5387)
Supplement: Supplementary file 1 — Supplementary Text Figs. S1 to S6 Table S1 References [file sciadv.abo5387_sm.pdf]

Science Advances
AAAS

Supplementary Materials for

Protein diffusion in *Escherichia coli* cytoplasm scales with the mass of the complexes and is location dependent

Wojciech M. Śmigiel *et al.*

Corresponding author: Bert Poolman, b.poolman@rug.nl

Sci. Adv. **8**, eabo5387 (2022)
DOI: 10.1126/sciadv.abo5387

The PDF file includes:

Supplementary Text
Figs. S1 to S6
Table S1
Legends for tables S2 and S3
References

Other Supplementary Materials for this manuscript includes the following:

Tables S2 and S3

Other Supplementary Materials for this manuscript includes:

- Table S2: Lengths and widths of analyzed cells.

  This table reports information on the length and the width of all the analyze cells. Cells' lengths and widths are separated in columns according to the protein they were expressing.

- Table S3: Diffusion coefficients and displacements of analyzed cells.

  This table reports information on the number of displacements measured in each region (cell center, right pole, left pole) of each cell and the related diffusion coefficient. Diffusion values and number of displacements are separated in different columns, representing the different regions of the cell. Data are then further separated in cluster representing the expressed protein.

Supplementary Text

On cytoplasmic viscosity

We observe that the diffusion coefficient scales with the complex molecular mass in the following manner:

$D_{observed} \approx \alpha M_{complex}^{-0.54}$

Where $M_{complex}$ represents the complex molecular mass, which was calculated as sum of the mass of the monomeric protein plus the fluorescent probe (mEos3.2) and multiplied by the oligomeric state number.

The Einstein-Stokes equation states that for non-interacting spherical particles moving of Brownian motion:

$$D = \frac{k_B T}{6\pi\eta r} \quad (1)$$

where k_B is the Boltzmann constant, T is the absolute temperature, η is the viscosity of the solvent and r is the radius of the diffusing particle. We can rewrite this equation as:

$$D = \frac{k_B T}{6\pi\eta R} = \frac{k_B T}{6\pi\eta\sqrt[3]{\frac{3}{4\pi}V_{sphere}}} \quad (2)$$

We know that:

$$V = \frac{m}{\rho} \quad (3)$$

$$m = moles * M_{complex} \quad (4)$$

$$1\,mole \equiv 1\,N_A\,of\,particles \quad (5)$$

Hence, we obtain:

$$V = \frac{M_{complex}}{\rho N_A} \quad (6)$$

So, we can rewrite the Einstein-Stokes equation as:

$$D \sim \frac{k_B T}{6\pi\eta\sqrt[3]{\frac{3}{4\pi}\frac{M_{complex}}{\rho N_A}}} \quad (7)$$

which means that:

$$D \propto M_{complex}^{-0.33} \quad (8)$$

Our experimental relationship differs substantially from the Einstein-Stokes equation. What is the basis for this difference? Clearly, the proteins are not perfect spheres, but if this parameter is important we would not have found the relationship with complex molecular mass. A similar argument could be made for surface charge. If deviations from the Einstein-Stokes equation are due to differences in protein charge, we would have lost the observed relationship. Another possibility would be that random walk is not an appropriate model for describing the diffusion of

proteins inside the bacterial cell. However we do not see any clear dependence of diffusion on any of the analyzed parameters (abundance, loneliness or oligomeric state) except for the complex mass. If random walk was not an appropriate model for diffusing particles in the bacterial cytoplasm, then we should have seen some relationship with one of the other analyzed parameters.

To ensure that our results were not biased by a systematic error, we compared a regression model with unconstrained fitting parameters and with an exponent power constrained to -0.33, which is the value given by the Einstein-Stokes equation. According to the extra sum-of-squares F test, which compares the goodness-of-fit of two alternative nested models (unconstrained and constrained), the unconstrained model with an exponent value of -0.54 was significantly better (p-value << 0.01) than the constrained model for describing the relationship between apparent diffusion and complex molecular weight for the set of native proteins. As a quantitative measure of fitting error for unconstrained and constrained regression models, the absolute sum of squares was selected. The absolute sum of squares for the unconstrained and constrained models are 741.4 and 1059.1, respectively, which indicates a 43% decrease in the deviation for the unconstrained fitting model. Hence, we conclude that the power-law model with an exponent of -0.54 is a better fit for correlating experimental diffusion data with the molecular weight of the protein complexes. We also notice that the protein density is more or less constant for proteins bigger than 30 kDa, with a fixed value of ~1.41 g/cm^3 as reported by Fischer et al. (*71*). Hence, this parameter is not a factor in the scaling of the diffusion coefficients of proteins. We hypothesize that the cell cytoplasm is a dilatant, non-Newtonian fluid, in which the viscosity increases as a function of the stress applied to the environment. Bigger particles diffusing in the solution will impose a higher stress compared to the smaller particles, which in turn will make the environment more viscous. By rearranging the Einstein-Stokes equation, we compare the perceived viscosity with the macromolecular size of the particles as a function of the observed diffusion coefficients:

$$\eta \sim \frac{k_B T}{6\pi D_{observed} \sqrt[3]{\frac{3}{4\pi} \frac{M_{complex}}{\rho N_A}}} \quad (9)$$

Hence, the perceived viscosity in the cytoplasm of *E. coli* can be written as a function of the mass of the macromolecules:

$$\eta_{perceived} \sim \alpha {M_{complex}}^{\beta} \quad (10)$$

If we want to obtain the relation of Equation 8, given the formula reported in equation 7, we will have that

$$\beta = -0.333 - (-0.535) = 0.202 \quad (11)$$

Hence:

$$\eta_{perceived} \sim \alpha {M_{complex}}^{0.20} \quad (12)$$

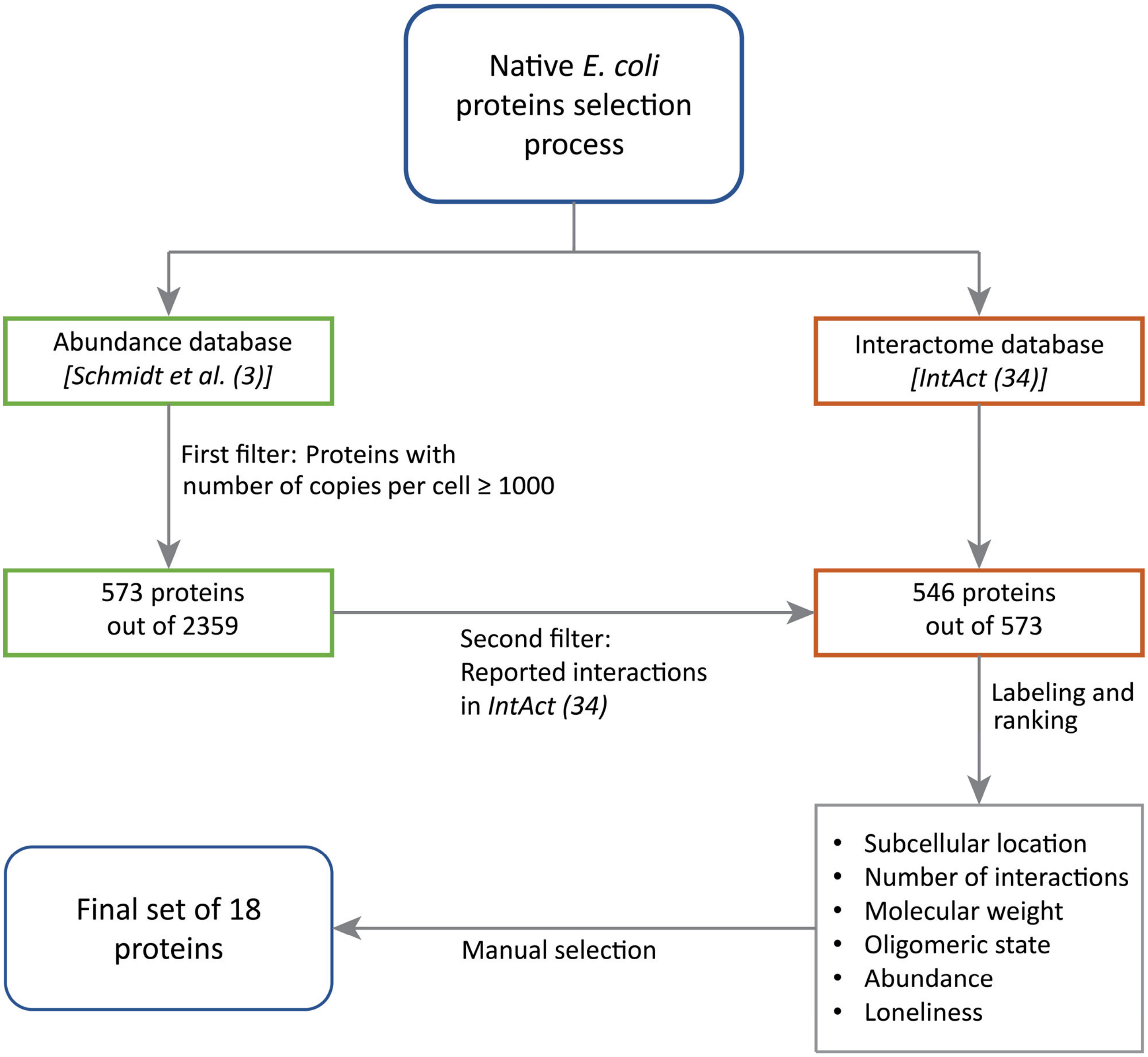

Fig. S1.

Schematic of the target selection process. The native *E. coli* proteins selected for SMdM analysis are specified in Supplementary Table S1.

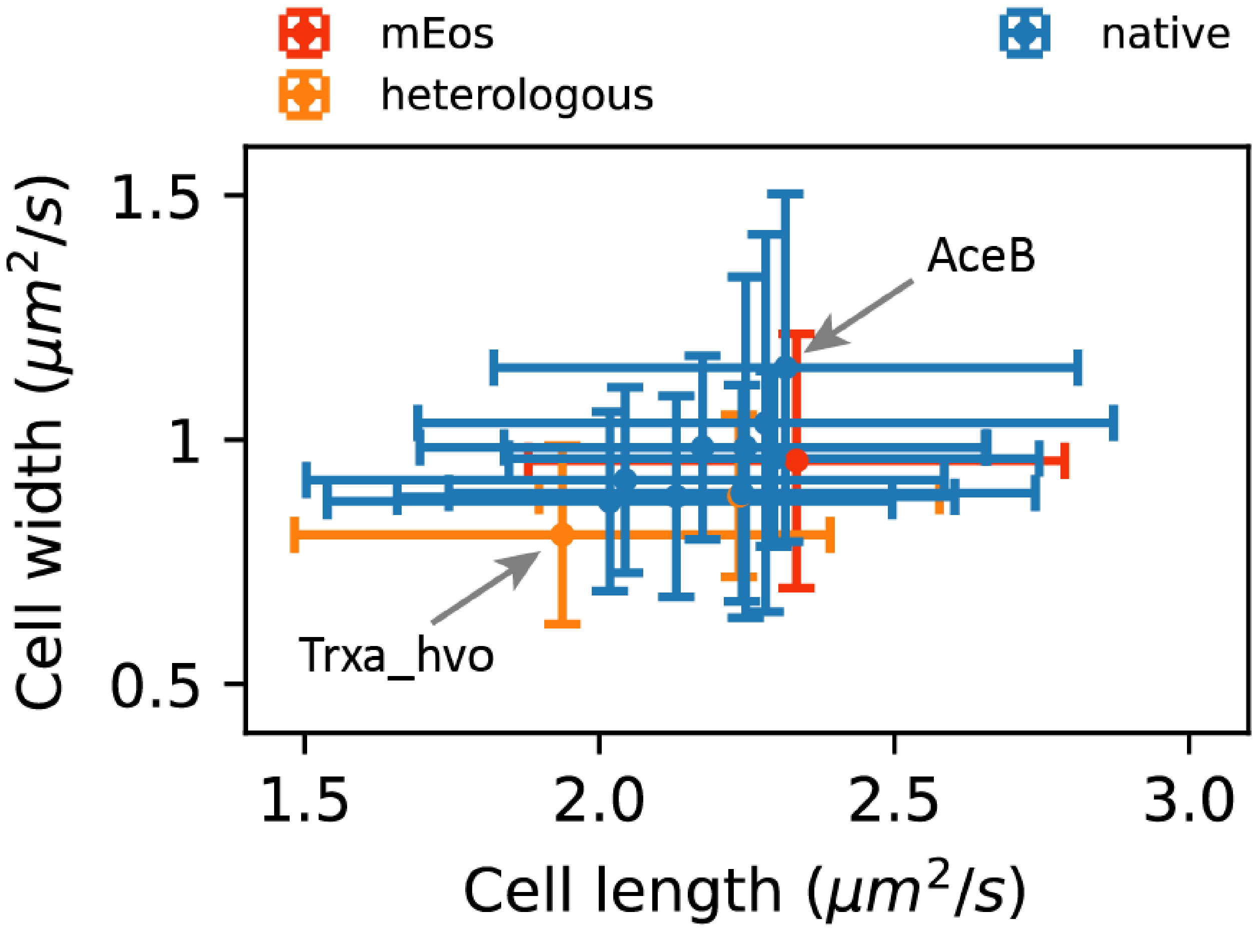

Fig. S2.

Comparison of cell width and cell length for each expression construct. The mean values are represented by dots; the error bars represent the standard deviation.

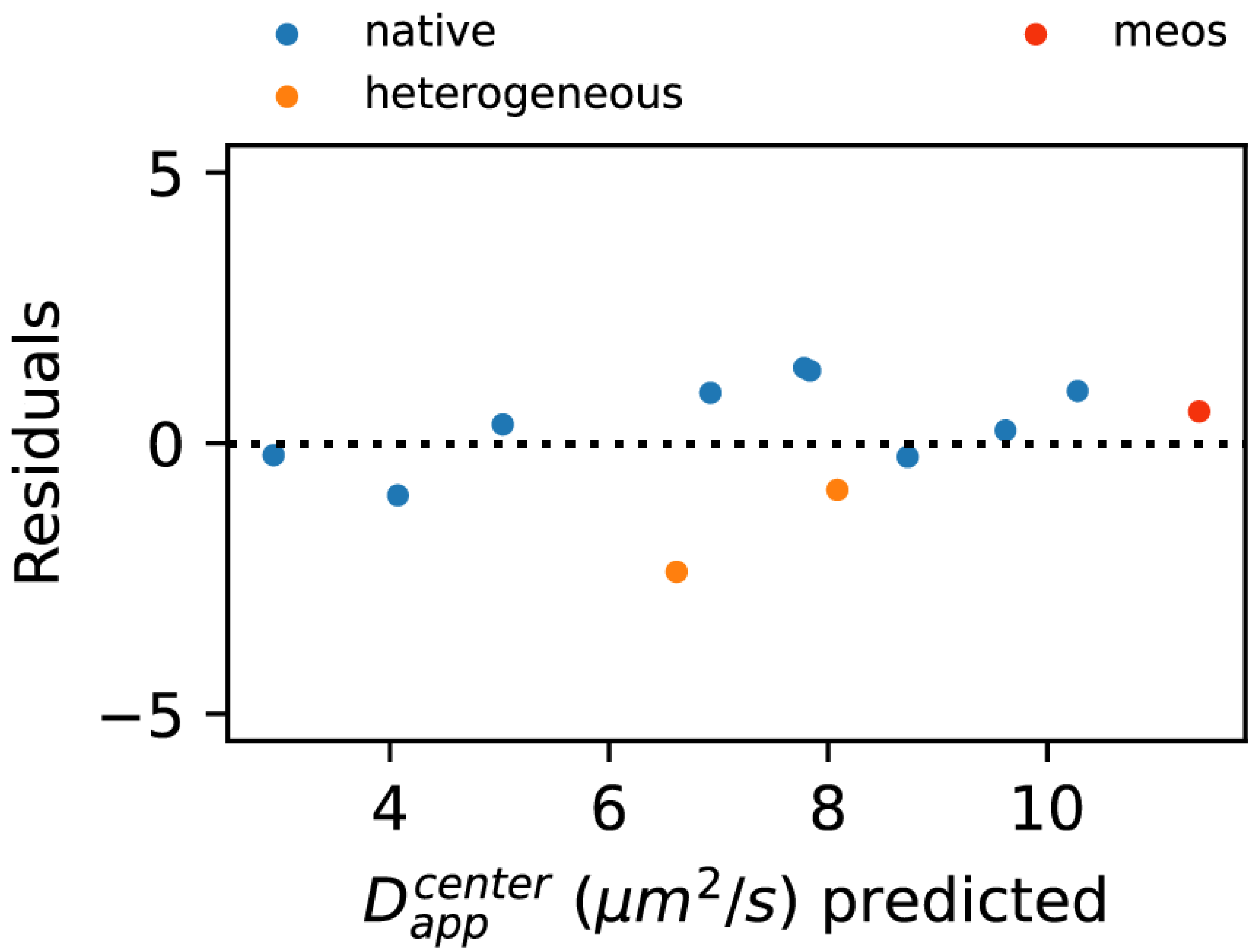

Fig. S3.

Analysis of the residuals from the plots in Figure 6D. The analysis shows no signs of correlation for the residuals. The diffusion coefficients of the heterologous proteins appear to be outliers when compared to the dataset obtained from only native proteins.

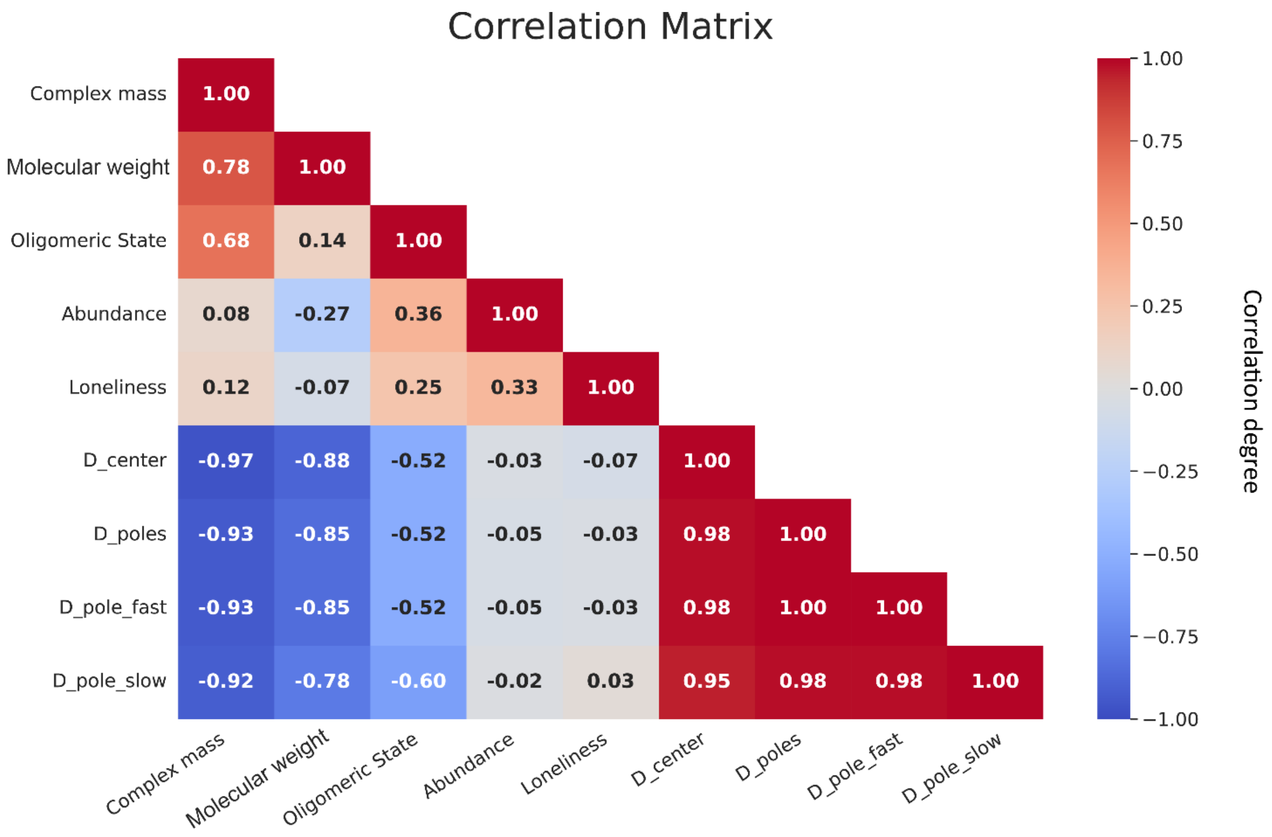

Fig. S4.

Multi-parameter analysis. Each variable has been tested for correlation with every other variable using the Spearman's rank correlation test. No correlation was found between loneliness and molecular weight, loneliness and abundance, and abundance and molecular weight. A moderate correlation between molecular weight and complex mass and between oligomeric and complex mass is observed, which is expected as the complex mass is calculated as the sum of the molecular weight of the monomeric protein plus fluorescence reporter and multiplied by the oligomeric state number. No correlation is observed between molecular weight and oligomeric state.

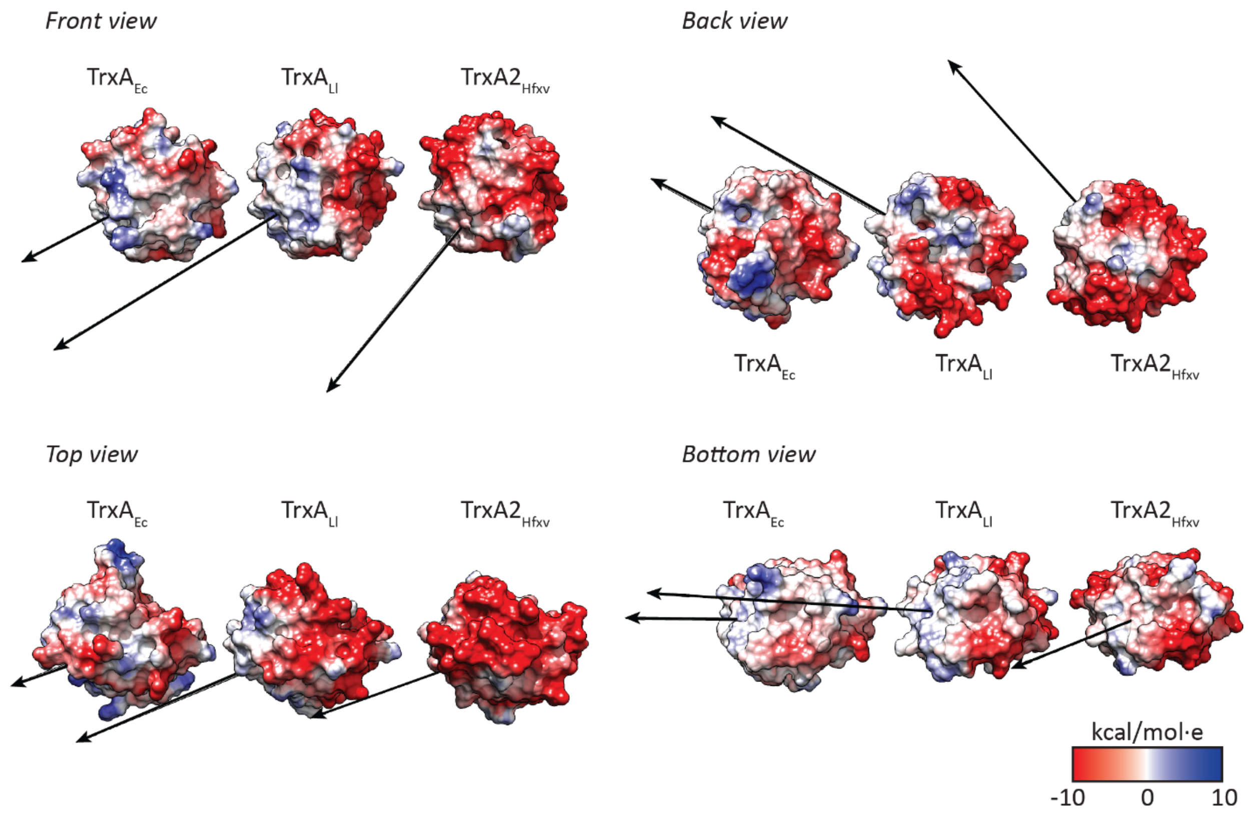

Fig. S5.

Different views of the surface charge distribution of the three homologous TrxA proteins. Top view corresponds to Fig. 5A.

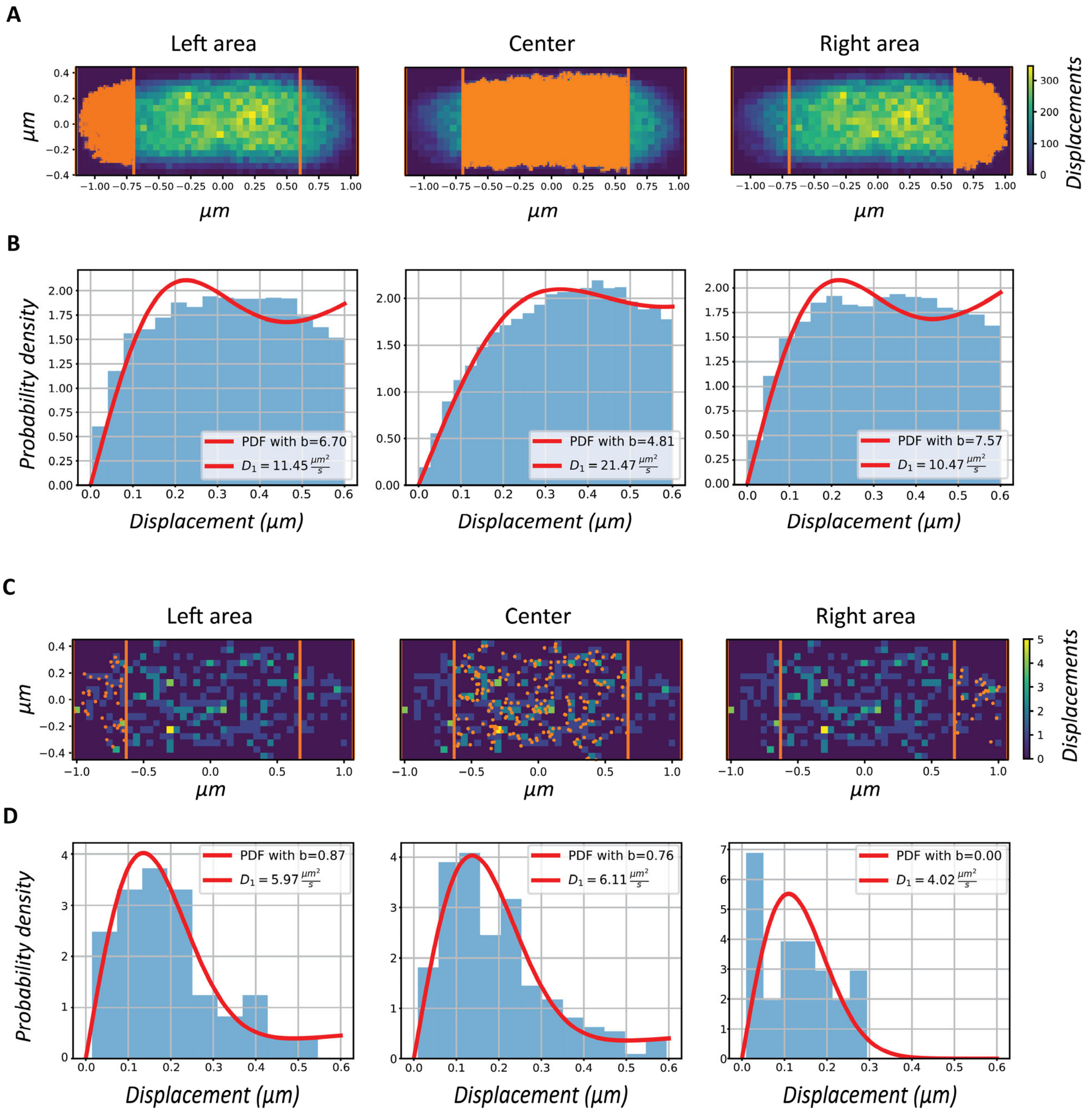

Fig. S6.

Examples of cells discarded from the analysis. A) Cell having too many displacements for being analyzed. B) Fitting of the cell in panel B. Too many displacements result in a poor fitting due to the high amount of background fluorescence. C) Cell not having enough displacements for being analyzed. D) Fitting of the cell in panel D. Too little displacements result in a poor fitting, which is especially evident for the fit of the right area.

Construct ID	UNIPROT ID	Protein name	MW (kDa)	Oligomeric state	Abundance (copies/cell)	Loneliness	comment
2	P0A9G6	AceA*	47.5	4	74908	47.2	aggregation
3	P00934	ThrC	47.1	1	11109	0.35	--
4	P18843	NadE*	30.6	2	3084	0.0037	aggregation
5	P0A836	SucC*	41.3	2/4	11673	0.037	heterotetramer
6	P0A9K9	SlyD*	20.8	1	15259	0.032	partial aggregation
7	P0C0L2	OsmC*	15.1	2	8157	0.78	aggregation
8	P0AC62	GrxC	9.1	1	6170	89.4	--
9	P05793	IlvC	54.0	4	29065	36.2	--
10	P00864	Ppc*	99.0	4	2185	10.02	failed to clone
11	P08997	AceB	60.2	1	8308	10.4	--
12	P0A6A8	AcpP	8.6	1	28863	0.12	--
13	P0ACC3	ErpA	12.1	2	3460	0.1	--
14	P0A763	Ndk*	15.4	4	17326	0.42	aggregation
15	P0AA25	TrxA	11.8	1	18242	0.025	--
16	P07813	LeuS	97.2	1	1505	0.005	--
17	P30125	LeuB*	39.5	2	5032	9.5	partial aggregation
18	P0A817	MetK*	41.9	4	6822	0.022	aggregation
19	P08200	Icd	45.7	2	24591	1.02	--

Table S1.

Final set of proteins chosen for SMdM analysis. Most of the proteins were successfully expressed as C-terminal fusions with mEos3.2. We noted the following exceptions: SlyD and LeuB expression yielded a mix of aggregating and non-aggregating cells; OsmC, Ndk, NadE, AceA, MetK displayed polar aggregation after overexpression in the majority of their cells; SucC forms an obligatory heterotetrameric complex with SucD; and Ppc failed to produce an expressing clone. Proteins marked with an asterisk were excluded from diffusion mapping. MW is the molecular weight (M_w) of a single chain; oligomeric state is according to the UNIPROT entry; abundance in copies per cell was taken from Schmidt et al. *(3)* for cells grown in M9-glycerol.

REFERENCES AND NOTES

1. S. H. Peeters, L. van Niftrik, Trending topics and open questions in anaerobic ammonium oxidation. *Curr. Opin. Chem. Biol.* **49**, 45–52 (2019).

2. M. Mori, Z. Zhang, A. Banaei-Esfahani, J.-B. Lalanne, H. Okano, B. C. Collins, A. Schmidt, O. T. Schubert, D.-S. Lee, G.-W. Li, R. Aebersold, T. Hwa, C. Ludwig, From coarse to fine: The absolute *Escherichia coli* proteome under diverse growth conditions. *Mol. Syst. Biol.* **17**, e9536 (2021).

3. A. Schmidt, K. Kochanowski, S. Vedelaar, E. Ahrné, B. Volkmer, L. Callipo, K. Knoops, M. Bauer, R. Aebersold, M. Heinemann, The quantitative and condition-dependent *Escherichia coli* proteome. *Nat. Biotechnol.* **34**, 104–110 (2016).

4. J. T. Mika, G. van den Bogaart, L. Veenhoff, V. Krasnikov, B. Poolman, Molecular sieving properties of the cytoplasm of *Escherichia coli* and consequences of osmotic stress. *Mol. Microbiol.* **77**, 200–207 (2010).

5. M. Kumar, M. S. Mommer, V. Sourjik, Mobility of cytoplasmic, membrane, and DNA-binding proteins in *Escherichia coli*. *Biophys. J.* **98**, 552–559 (2010).

6. F. D. Schramm, K. Schroeder, K. Jonas, Protein aggregation in bacteria. *FEMS Microbiol. Rev.* **44**, 54–72 (2020).

7. A.-S. Coquel, J.-P. Jacob, M. Primet, A. Demarez, M. Dimiccoli, T. Julou, L. Moisan, A. B. Lindner, H. Berry, Localization of protein aggregation in *Escherichia coli* is governed by diffusion and nucleoid macromolecular crowding effect. *PLoS Comput. Biol.* **9**, e1003038 (2013).

8. S. A. Keatch, P. G. Leonard, J. E. Ladbury, D. T. F. Dryden, StpA protein from *Escherichia coli* condenses supercoiled DNA in preference to linear DNA and protects it from digestion by DNase I and EcoKI. *Nucleic Acids Res.* **33**, 6540–6546 (2005).

9. P. E. Schavemaker, W. M. Śmigiel, B. Poolman, Ribosome surface properties may impose limits on the nature of the cytoplasmic proteome. *eLife* **6**, e30084 (2017).

10. H. Shi, B. P. Bratton, Z. Gitai, K. C. Huang, How to build a bacterial cell: MreB as the foreman of *E. coli* construction. *Cell* **172**, 1294–1305 (2018).

11. S. Bakshi, A. Siryaporn, M. Goulian, J. C. Weisshaar, Superresolution imaging of ribosomes and RNA polymerase in live *Escherichia coli* cells. *Mol. Microbiol.* **85**, 21–38 (2012).

12. S. Bakshi, H. Choi, J. C. Weisshaar, The spatial biology of transcription and translation in rapidly growing *Escherichia coli*. *Front. Microbiol.* **6**, 636 (2015).

13. W. T. Gray, S. K. Govers, Y. Xiang, B. R. Parry, M. Campos, S. Kim, C. Jacobs-Wagner, Nucleoid size scaling and intracellular organization of translation across bacteria. *Cell* **177**, 1632–1648.e20 (2019).

14. G. Laloux, C. Jacobs-Wagner, How do bacteria localize proteins to the cell pole? *J. Cell Sci.* **127**, 11–19 (2014).

15. B. R. Parry, I. V. Surovtsev, M. T. Cabeen, C. S. O'Hern, E. R. Dufresne, C. Jacobs-Wagner, The bacterial cytoplasm has glass-like properties and is fluidized by metabolic activity. *Cell* **156**, 183–194 (2014).

16. K. Nishizawa, K. Fujiwara, M. Ikenaga, N. Nakajo, M. Yanagisawa, D. Mizuno, Universal glass-forming behavior of in vitro and living cytoplasm. *Sci. Rep.* **7**, 15143 (2017).

17. R. P. Joyner, J. H. Tang, J. Helenius, E. Dultz, C. Brune, L. J. Holt, S. Huet, D. J. Müller, K. Weis, A glucose-starvation response regulates the diffusion of macromolecules. *eLife* **5**, e09376 (2016).

18. M. C. Munder, D. Midtvedt, T. Franzmann, E. Nüske, O. Otto, M. Herbig, E. Ulbricht, P. Müller, A. Taubenberger, S. Maharana, L. Malinovska, D. Richter, J. Guck, V. Zaburdaev, S. Alberti, A pH-driven transition of the cytoplasm from a fluid- to a solid-like state promotes entry into dormancy. *eLife* **5**, e09347 (2016).

19. B. Guilhas, J.-C. Walter, J. Rech, G. David, N. O. Walliser, J. Palmeri, C. Mathieu-Demaziere, A. Parmeggiani, J.-Y. Bouet, A. Le Gall, M. Nollmann, ATP-driven separation of liquid phase condensates in bacteria. *Mol. Cell* **79**, 293–303.e4 (2020).

20. L. Babl, G. Giacomelli, B. Ramm, A.-K. Gelmroth, M. Bramkamp, P. Schwille, CTP-controlled liquid–liquid phase separation of ParB. *J. Mol. Biol.* **434**, 167401 (2022).

21. X. Jin, J.-E. Lee, C. Schaefer, X. Luo, A. J. M. Wollman, A. L. Payne-Dwyer, T. Tian, X. Zhang, X. Chen, Y. Li, T. C. B. McLeish, M. C. Leake, F. Bai, Membraneless organelles formed by liquid-liquid phase separation increase bacterial fitness. *Sci. Adv.* **7**, eabh2929 (2021).

22. A.-M. Ladouceur, B. S. Parmar, S. Biedzinski, J. Wall, S. G. Tope, D. Cohn, A. Kim, N. Soubry, R. Reyes-Lamothe, S. C. Weber, Clusters of bacterial RNA polymerase are biomolecular condensates that assemble through liquid–liquid phase separation. *Proc. Natl. Acad. Sci. U.S.A.* **117**, 18540–18549 (2020).

23. V. Norris, T. den Blaauwen, A. Cabin-Flaman, R. H. Doi, R. Harshey, L. Janniere, A. Jimenez-Sanchez, D. J. Jin, P. A. Levin, E. Mileykovskaya, A. Minsky, M. Saier Jr., K. Skarstad, Functional taxonomy of bacterial hyperstructures. *Microbiol. Mol. Biol. Rev.* **71**, 230–253 (2007).

24. C. A. Azaldegui, A. G. Vecchiarelli, J. S. Biteen, The emergence of phase separation as an organizing principle in bacteria. *Biophys. J.* **120**, 1123–1138 (2021).

25. M. L. Heltberg, J. Miné-Hattab, A. Taddei, A. M. Walczak, T. Mora, Physical observables to determine the nature of membrane-less cellular sub-compartments. *eLife* **10**, e69181 (2021).

26. L. Hubatsch, L. M. Jawerth, C. Love, J. Bauermann, T. D. Tang, S. Bo, A. A. Hyman, C. A. Weber, Quantitative theory for the diffusive dynamics of liquid condensates. *eLife* **10**, e68620 (2021).

27. Y. Xiang, I. V. Surovtsev, Y. Chang, S. K. Govers, B. R. Parry, J. Liu, C. Jacobs-Wagner, Interconnecting solvent quality, transcription, and chromosome folding in *Escherichia coli*. *Cell* **184**, 3626–3642.e14 (2021).

28. J. T. Mika, B. Poolman, Macromolecule diffusion and confinement in prokaryotic cells. *Curr. Opin. Biotechnol.* **22**, 117–126 (2011).

29. J. Spitzer, B. Poolman, How crowded is the prokaryotic cytoplasm? *FEBS Lett.* **587**, 2094–2098 (2013).

30. J. Spitzer, B. Poolman, The role of biomacromolecular crowding, ionic strength, and physicochemical gradients in the complexities of life's emergence. *Microbiol. Mol. Biol. Rev.* **73**, 371–388 (2009).

31. J. van den Berg, A. J. Boersma, B. Poolman, Microorganisms maintain crowding homeostasis. *Nat. Rev. Microbiol.* **15**, 309–318 (2017).

32. M. Zhang, H. Chang, Y. Zhang, J. Yu, L. Wu, W. Ji, J. Chen, B. Liu, J. Lu, Y. Liu, J. Zhang, P. Xu, T. Xu, Rational design of true monomeric and bright photoactivatable fluorescent proteins. *Nat. Methods* **9**, 727–729 (2012).

33. L. Xiang, K. Chen, R. Yan, W. Li, K. Xu, Single-molecule displacement mapping unveils nanoscale heterogeneities in intracellular diffusivity. *Nat. Methods* **17**, 524–530 (2020).

34. S. Orchard, M. Ammari, B. Aranda, L. Breuza, L. Briganti, F. Broackes-Carter, N. H. Campbell, G. Chavali, C. Chen, N. del-Toro, M. Duesbury, M. Dumousseau, E. Galeota, U. Hinz, M. Iannuccelli, S. Jagannathan, R. Jimenez, J. Khadake, A. Lagreid, L. Licata, R. C. Lovering, B. Meldal, A. N. Melidoni, M. Milagros, D. Peluso, L. Perfetto, P. Porras, A. Raghunath, S. Ricard-Blum, B. Roechert, A. Stutz, M. Tognolli, K. van Roey, G. Cesareni, H. Hermjakob, The MIntAct project—IntAct as a common curation platform for 11 molecular interaction databases. *Nucleic Acids Res.* **42**, D358–D363 (2014).

35. B. P. English, V. Hauryliuk, A. Sanamrad, S. Tankov, N. H. Dekker, J. Elf, Single-molecule investigations of the stringent response machinery in living bacterial cells. *Proc. Natl. Acad. Sci. U.S.A.* **108**, E365–E373 (2011).

36. S. S. Andrews, Smoldyn: Particle-based simulation with rule-based modeling, improved molecular interaction and a library interface. *Bioinformatics* **33**, 710–717 (2017).

37. F. B. Knight, On the random walk and Brownian motion. *Trans. Amer. Math. Soc.* **103**, 218–228 (1962).

38. G. Crivat, J. W. Taraska, Imaging proteins inside cells with fluorescent tags. *Trends Biotechnol*. **30**, 8–16 (2012).

39. E. C. Jensen, Use of fluorescent probes: Their effect on cell biology and limitations. *Anat. Rec.* **295**, 2031–2036 (2012).

40. L. Mantovanelli, B. F. Gaastra, B. Poolman, Fluorescence-based sensing of the bioenergetic and physicochemical status of the cell, in *Current Topics in Membranes*: *New Methods and Sensors for Membrane and Cell Volume Research*, M. A. Model, I. Levitan, Eds. (Academic Press, 2021), vol. 88, pp. 1–54.

41. A. Patel, L. Malinovska, S. Saha, J. Wang, S. Alberti, Y. Krishnan, A. A. Hyman, ATP as a biological hydrotrope. *Science* **356**, 753–756 (2017).

42. A. Nenninger, G. Mastroianni, C. W. Mullineaux, Size dependence of protein diffusion in the cytoplasm of *Escherichia coli*. *J. Bacteriol.* **192**, 4535–4540 (2010).

43. P. E. Schavemaker, A. J. Boersma, B. Poolman, How important is protein diffusion in prokaryotes? *Front. Mol. Biosci.* **5**, 93 (2018).

44. E. O. Puchkov, Intracellular viscosity: Methods of measurement and role in metabolism. *Biochem. Moscow Suppl. Ser. A.* **7**, 270–279 (2013).

45. UniProt Consortium, UniProt: The universal protein knowledgebase in 2021. *Nucleic Acids Res.* **49**, D480–D489 (2021).

46. MembraneEnzymology, *MembraneEnzymology/smdm: SMdM analysis in Escherichia coli cytoplasm* (Zenodo, 2022); https://zenodo.org/record/5911836.

47. S. Kerrien, S. Orchard, L. Montecchi-Palazzi, B. Aranda, A. F. Quinn, N. Vinod, G. D. Bader, I. Xenarios, J. Wojcik, D. Sherman, M. Tyers, J. J. Salama, S. Moore, A. Ceol, A. Chatr-aryamontri, M. Oesterheld, V. Stümpflen, L. Salwinski, J. Nerothin, E. Cerami, M. E. Cusick, M. Vidal, M. Gilson, J. Armstrong, P. Woollard, C. Hogue, D. Eisenberg, G. Cesareni, R. Apweiler, H. Hermjakob, Broadening the horizon – level 2.5 of the HUPO-PSI format for molecular interactions. *BMC Biol.* **5**, 44 (2007).

48. M. Ashburner, C. A. Ball, J. A. Blake, D. Botstein, H. Butler, J. M. Cherry, A. P. Davis, K. Dolinski, S. S. Dwight, J. T. Eppig, M. A. Harris, D. P. Hill, L. Issel-Tarver, A. Kasarskis, S. Lewis, J. C. Matese, J. E. Richardson, M. Ringwald, G. M. Rubin, G. Sherlock, Gene Ontology: Tool for the unification of biology. *Nat. Genet.* **25**, 25–29 (2000).

49. Gene Ontology Consortium, The Gene Ontology resource: Enriching a GOld mine. *Nucleic Acids Res.* **49**, D325–D334 (2021).

50. F. Geu-Flores, H. H. Nour-Eldin, M. T. Nielsen, B. A. Halkier, USER fusion: A rapid and efficient method for simultaneous fusion and cloning of multiple PCR products. *Nucleic Acids Res.* **35**, e55 (2007).

1. R. Green, E. J. Rogers, in *Methods in Enzymology*, vol. 529, *Laboratory Methods in Enzymology: DNA*, J. Lorsch, Ed. (Academic Press, 2013), pp. 329–336.

52. G. Bertani, Studies on lysogenesis. I. The mode of phage liberation by lysogenic *Escherichia coli*. *J. Bacteriol.* **62**, 293–300 (1951).

53. F. C. Neidhardt, P. L. Bloch, D. F. Smith, Culture medium for enterobacteria. *J. Bacteriol.* **119**, 736–747 (1974).

54. M. Tokunaga, N. Imamoto, K. Sakata-Sogawa, Highly inclined thin illumination enables clear single-molecule imaging in cells. *Nat. Methods* **5**, 159–161 (2008).

55. H. Babcock, Y. M. Sigal, X. Zhuang, A high-density 3D localization algorithm for stochastic optical reconstruction microscopy. *Opt. Nanoscopy* **1**, 6 (2012).

56. D. R. Edla, P. K. Jana, A novel clustering algorithm using Voronoi diagram, in *Seventh International Conference on Digital Information Management (ICDIM)* (Macau, 2012), pp. 35–40.

57. P. Virtanen, R. Gommers, T. E. Oliphant, M. Haberland, T. Reddy, D. Cournapeau, E. Burovski, P. Peterson, W. Weckesser, J. Bright, S. J. van der Walt, M. Brett, J. Wilson, K. J. Millman, N. Mayorov, A. R. J. Nelson, E. Jones, R. Kern, E. Larson, C. J. Carey, İ. Polat, Y. Feng, E. W. Moore, J. V. Plas, D. Laxalde, J. Perktold, R. Cimrman, I. Henriksen, E A Quintero, C. R. Harris, A. M. Archibald, A. H. Ribeiro, F. Pedregosa, P. van Mulbregt; SciPy 1.0 Contributors, SciPy 1.0: Fundamental algorithms for scientific computing in Python. *Nat. Methods* **17**, 261–272 (2020).

58. I. J. Myung, Tutorial on maximum likelihood estimation. *J. Math. Psychol.* **47**, 90–100 (2003).

59. J. Reback, jbrockmendel, W. McKinney, J. Van den Bossche, T. Augspurger, P. Cloud, S. Hawkins, M. Roeschke, gfyoung, Sinhrks, A. Klein, P. Hoefler, T. Petersen, J. Tratner, C. She, W. Ayd, S. Naveh, M. Garcia, J. H. M. Darbyshire, J. Schendel, R. Shadrach, A. Hayden, D. Saxton, M. E.

Gorelli, F. Li, M. Zeitlin, V. Jancauskas, A. McMaster, P. Battiston, S. Seabold, *pandas-dev/pandas: Pandas 1.4.0* (Zenodo, 2022); https://zenodo.org/record/5893288.

60. C. R. Harris, K. J. Millman, S. J. van der Walt, R. Gommers, P. Virtanen, D. Cournapeau, E. Wieser, J. Taylor, S. Berg, N. J. Smith, R. Kern, M. Picus, S. Hoyer, M. H. van Kerkwijk, M. Brett, A. Haldane, J. F. del Río, M. Wiebe, P. Peterson, P. Gérard-Marchant, K. Sheppard, T. Reddy, W. Weckesser, H. Abbasi, C. Gohlke, T. E. Oliphant, Array programming with NumPy. *Nature* **585**, 357–362 (2020).

61. N. Guex, M. C. Peitsch, T. Schwede, Automated comparative protein structure modeling with SWISS-MODEL and Swiss-PdbViewer: A historical perspective. *Electrophoresis* **30**, S162–S173 (2009).

2. A. Waterhouse, M. Bertoni, S. Bienert, G. Studer, G. Tauriello, R. Gumienny, F. T. Heer, T. A. P. de Beer, C. Rempfer, L. Bordoli, R. Lepore, T. Schwede, SWISS-MODEL: Homology modelling of protein structures and complexes. *Nucleic Acids Res.* **46**, W296–W303 (2018).

63. L. Corsini, M. Hothorn, G. Stier, V. Rybin, K. Scheffzek, T. J. Gibson, M. Sattler, Dimerization and protein binding specificity of the U2AF homology motif of the splicing factor Puf60. *J. Biol. Chem.* **284**, 630–639 (2009).

4. G. Roos, A. Garcia-Pino, K. Van Belle, E. Brosens, K. Wahni, G. Vandenbussche, L. Wyns, R. Loris, J. Messens, The conserved active site proline determines the reducing power of *Staphylococcus aureus* thioredoxin. *J. Mol. Biol.* **368**, 800–811 (2007).

65. S. Arai, C. Shibazaki, R. Shimizu, M. Adachi, M. Ishibashi, H. Tokunaga, M. Tokunaga, Catalytic mechanism and evolutionary characteristics of thioredoxin from Halobacterium salinarum NRC-1. *Acta Crystallogr. D Struct. Biol.* **76**, 73–84 (2020).

66. E. F. Pettersen, T. D. Goddard, C. C. Huang, G. S. Couch, D. M. Greenblatt, E. C. Meng, T. E. Ferrin, UCSF Chimera—A visualization system for exploratory research and analysis. *J. Comput. Chem.* **25**, 1605–1612 (2004).

67. C. Spearman, Spearman rank correlation coefficient, in *The Concise Encyclopedia of Statistics* (Springer, 2008), pp. 502–505; https://doi.org/10.1007/978-0-387-32833-1_379.

68. S. S. Shapiro, M. B. Wilk, An analysis of variance test for normality (complete samples). *Biometrika* **52**, 591–611 (1965).

69. P. E. McKnight, J. Najab, Mann-Whitney U test, in *The Corsini Encyclopedia of Psychology* (John Wiley & Sons Ltd, 2010), pp. 1–1; https://onlinelibrary.wiley.com/doi/abs/10.1002/9780470479216.corpsy0524.

70. S. J. Sheather, Density estimation. *Stat. Sci.* **19**, 588–597 (2004).

71. H. Fischer, I. Polikarpov, A. F. Craievich, Average protein density is a molecular-weight-dependent function. *Protein Sci.* **13**, 2825–2828 (2004).
